# Surgical polarimetric endoscopy for the detection of laryngeal cancer

**DOI:** 10.1038/s41551-023-01018-0

**Published:** 2023-04-03

**Authors:** Ji Qi, Taranjit Tatla, Eranga Nissanka-Jayasuriya, Alan Yilun Yuan, Danail Stoyanov, Daniel S. Elson

**Affiliations:** 1 Research Center for Humanoid Sensing, Zhejiang Lab, Hangzhou, China; 2 Wellcome/EPSRC Centre for Interventional and Surgical Sciences, University College London, London, UK; 3 Department of Computer Science, University College London, London, UK; 4 Centre For Medical Image Computing, University College London, London, UK; 5 Hamlyn Centre for Robotic Surgery, Imperial College London, London, UK; 6 Department of Surgery and Cancer, Imperial College London, London, UK; 7 Northwick Park Hospital, London North West University Healthcare NHS Trust, London, UK; 8 William Harvey Hospital, East Kent Hospitals University NHS Foundation Trust, London, UK; 9 Department of Electrical and Electronic Engineering, Imperial College London, London, UK

**Keywords:** Endoscopy, Applied optics, Biomedical engineering

## Abstract

The standard-of-care for the detection of laryngeal pathologies involves distinguishing suspicious lesions from surrounding healthy tissue via contrasts in colour and texture captured by white-light endoscopy. However, the technique is insufficiently sensitive and thus leads to unsatisfactory rates of false negatives. Here we show that laryngeal lesions can be better detected in real time by taking advantage of differences in the light-polarization properties of cancer and healthy tissues. By measuring differences in polarized-light retardance and depolarization, the technique, which we named ‘surgical polarimetric endoscopy’ (SPE), generates about one-order-of-magnitude greater contrast than white-light endoscopy, and hence allows for the better discrimination of cancerous lesions, as we show with patients diagnosed with squamous cell carcinoma. Polarimetric imaging of excised and stained slices of laryngeal tissue indicated that changes in the retardance of polarized light can be largely attributed to architectural features of the tissue. We also assessed SPE to aid routine transoral laser surgery for the removal of a cancerous lesion, indicating that SPE can complement white-light endoscopy for the detection of laryngeal cancer.

## Abstract (web summary)

Laryngeal lesions can be endoscopically detected with high sensitivity and in real time by measuring differences in the polarization signatures between cancerous and healthy tissues.

## Main

Laryngeal cancer is one of the most common malignancies of the upper aerodigestive tract^1^. Minimally invasive procedures, including endoscopic incisional biopsy and transoral surgery, are widely used for laryngeal cancer diagnosis and intervention, respectively. Surgical endoscopy plays a key role by allowing surgeons to inspect suspicious areas in detail without having to invasively open and expose the larynx, and also to further decide where to biopsy for histopathological examination or where to resect in surgical intervention. The standard-of-care is high-definition white-light endoscopy (WLE), a wide-field optical imaging technique using white-light illumination and red/green/blue (RGB) colour camera imaging of larynx tissue via a surgical endoscope, closely recreating what would be seen by the naked eye. Surgeons rely on the WLE colour and texture contrast to differentiate suspicious laryngeal lesions from surrounding normal tissue and then perform the operation. Unfortunately, tumourous lesions—especially those at the pre-cancerous and early-cancerous stages—may not always present discernible colour or texture differences, while tissue regions with abnormal colour or texture may not always be associated with cancer, leading to unsatisfactory sensitivity (as low as 51.1%) and miss rate^2–5^. The limitations of WLE result in inappropriate selection of biopsy targets, or subtotal/unnecessary resection, negatively affecting survival rate or causing additional functional impairment for the patient.

Minimally invasive procedures for laryngeal cancer can benefit immensely by introducing complementary surgical endoscopy techniques that enhance the contrast associated with pathologies into the clinical workflow. The ideal technique would deliver a real-time, high-definition, wide-field and label-free imaging solution to aid rapid cancer detection and clear operative guidance^6^. There are several promising emerging biophotonics techniques including fluorescence confocal endo-microscopy and non-linear endo-microscopy for cellular level visualization of tissue^7–11^, endoscopic optical coherence tomography for tissue architecture identification^12–16^, endoscopic light scattering spectroscopy for cell morphology detection^17–19^ and so on. These use either point-like detection (along the lateral direction) or imaging within a submillimetre field of view and require additional ad hoc sampling or scanning mechanism within an endoscope system to cover a wider area. It is challenging to acquire high-definition images with reasonably wide area of coverage comparable to WLE or the naked eye in real time, which limits the ability of these techniques for detecting moving and deforming surgical scenes and causes challenges in registering the information extracted from these techniques with the surgeons’ view of tissue and surgical tools.

The development of polarimetric imaging allows high-definition, real-time, wide-field and label-free endoscopic imaging to enhance intra-operative laryngeal cancer detection. Although it has been reported that polarimetric imaging can provide useful information for colon, cervical and oesophageal cancer detection ex vivo^20–23^, there are major technical hurdles to improve imaging speed, instrument size and weight to permit intra-operative polarimetric imaging on human in vivo through an endoscope. Polarimetric imaging typically requires cumbersome polarization modulation devices, lengthy sequential radiometric image capture and time-consuming processing to reconstruct individual tissue polarimetric images. A lightweight and compact polarimetric endoscope with real-time performance suitable for in vivo intra-operative human study has not been demonstrated so far^23–32^. This has hindered translational research to assess the role of polarimetric imaging techniques for cancer detection and efforts to better understand the underlying origin of polarimetric contrast manifested in cancer pathologies.

In this Article, to bridge this gap, we demonstrate a new endoscopic imaging technique named surgical polarimetric endoscopy (SPE) that can achieve real-time image acquisition, reconstruction and display. We validated the SPE system by imaging phantoms with known polarization properties and showed the proof-of-concept of SPE via imaging of non-stationary target in vivo. A first-in-human study in the operating theatre was performed to assess the potential role of SPE in intra-operative laryngeal cancer detection, followed by histology and polarization microscopy validation. The results show that SPE offers useful polarimetric contrast not available in WLE to better differentiate laryngeal cancerous lesions from the normal tissue and reveal that the contrast arises from the tissue architectural changes. Our study indicates that this label-free, contact-free, wide-field, high-definition endoscopic imaging technique has translational potential and practicality to aid intra-operative laryngeal cancer detection and holds great promise to become an effective complementary method to standard-of-care WLE.

## Results

### Engineering a real-time SPE system

**Fig. 1:**
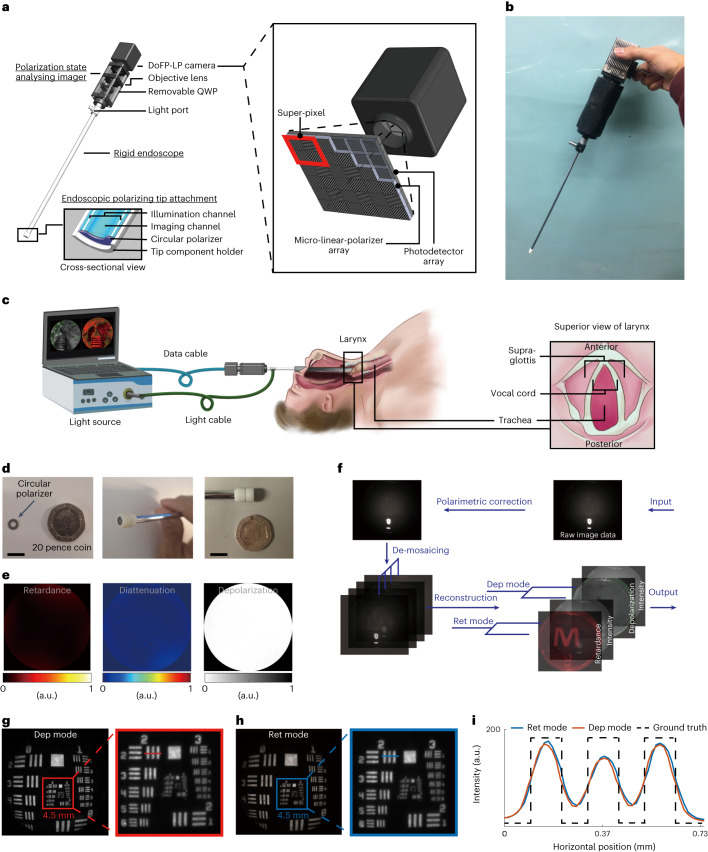
The SPE system. **a**, Design of the handheld endoscope device in the SPE system and schematic of DoFP-LP camera. QWP: quarter waveplate. DoFP-LP incorporates an array of micro-linear polarizers on the top of the photodiode array. Every block of four adjacent array elements comprises 90°, 45°, 135° and 0° angled micro-linear polarizers directly above four photodiodes that constitute a super-pixel. **b**, Photo of the handheld endoscope part of the SPE system. **c**, Schematic: intra-operative imaging of larynx with the SPE system. **d**, Endoscopic polarizing tip attachment, compared with a 20 pence coin. Scale bar is 10 mm. **e**, The polarization properties (from left to right: retardance, diattenuation and depolarization; unitless) of the imaging channel of the rigid endoscope used in the SPE system. **f**, Image processing pipeline. **g**,**h**, Intensity reference images obtained from SPE imaging of a negative 1951 USAF target under depolarization (dep) (**g**) and retardance (ret) (**h**) modes. **i**, The spread functions of a three-line grid in the target extracted from the red horizontal line in **g** and blue horizontal line in **h**. Source data

The SPE system consists of a handheld endoscope device (Fig. 1a,b) including an endoscopic polarizing tip attachment, a rigid endoscope, a polarization state analysing imager and a base station with an endoscope light source and a computer for data acquisition, reconstruction and display. The SPE base station was positioned on a portable clinical cart, while the handheld part was transorally introduced to the larynx by the surgeon (Fig. 1c). A full description of the system can be found in Methods.

With circularly polarized illumination, tissue linear retardance and circular depolarization can be obtained from efficient arithmetic operations on the Stokes vector (with four elements *S*_0_, *S*_1_, *S*_2_ and *S*_3_) of the backscattered light^33–36^. The SPE system therefore uses circular polarization for illumination, generated by the endoscopic tip attachment (Fig. 1d). However, the imaging channels of routine rigid endoscopes have intrinsic polarization properties that distort the polarization state of the incident light^25,37,38^, preventing polarization state analysis at the proximal end of a rigid endoscope, while miniaturization of a polarization state analyser to the distal end of the scope imposes a challenging size restriction. Instead, we sourced an endoscope with fully polarization maintaining imaging channel (diattenuation, depolarization and retardance are close to 0, Fig. 1e; its Mueller matrix image is available in Supplementary Fig. 1) that effectively enabled polarization state analysis without a strict size restriction at the proximal end of the endoscope.

To enable snapshot imaging, we incorporated a dual-imaging-mode design to allow the SPE system operate individually under either retardance or depolarization modes. A key enabling element of this design is a division-of-focal-plane linear polarimetric (DoFP-LP) image sensor (Fig. 1a), a commercially available device that incorporates an array of micro-linear polarizers made of aluminium nano-wire grids on the top of the photodiode array, which allows linear polarization states to be analysed in a single snapshot. In depolarization mode, a pre-aligned quarter waveplate is placed between the DoFP-LP camera and the eyepiece of the rigid endoscope so that *S*_0_ and *S*_3_ can be obtained in a single snapshot for simultaneous reconstruction of the depolarization and polarization insensitive radiometric intensity images. In retardance mode, the waveplate is removed to record *S*_0_, *S*_1_ and *S*_2_ in a snapshot for simultaneous recovery of retardance and the polarization insensitive radiometric intensity images (for full description, see Methods). The mode can be switched in less than 5 s by placing/removing the waveplate. The polarization insensitive radiometric intensity image obtained from SPE is equivalent to that acquired with conventional monochrome imaging, which thus serves as an important ‘intensity reference’ to guide intra-operative manipulation of the SPE and to interpret the reconstructed depolarization or retardance images.

To display the reconstructed images in real time, we developed a pipeline to process the output data from the SPE (Fig. 1f). This included polarimetric correction to address the inhomogeneity of the micro-polarizers of the DoFP-LP, de-mosaicing to reconstruct four linear polarization subimages from the interlaced micro-polarizer array, and reconstruction of retardance or depolarization image together with its intensity-reference image (Methods). The intensity-reference image may undergo optional gamma correction and sharpening to improve visibility.

The engineered SPE system has digital resolution 1,384 × 1,208 pixels, and a frame rate of 9.5 fps, with 53.3 ms exposure time and 52.0 ms processing time per frame for the in vivo experiments. If processing is not implemented online, the maximum frame rate of the SPE system is up to 35 fps with 28.6 ms exposure time. The SPE system has a standard circular endoscopic field of view with 104° angle of view. The optical resolution for both modes was 10.10 lp mm^−1^ characterized with a negative 1951 USAF target at 1 cm away from the tip of the endoscope (Fig. 1g,h). The spread function of a three-line grid in the target is shown in Fig. 1i.

### Technical validation of the SPE

**Fig. 2:**
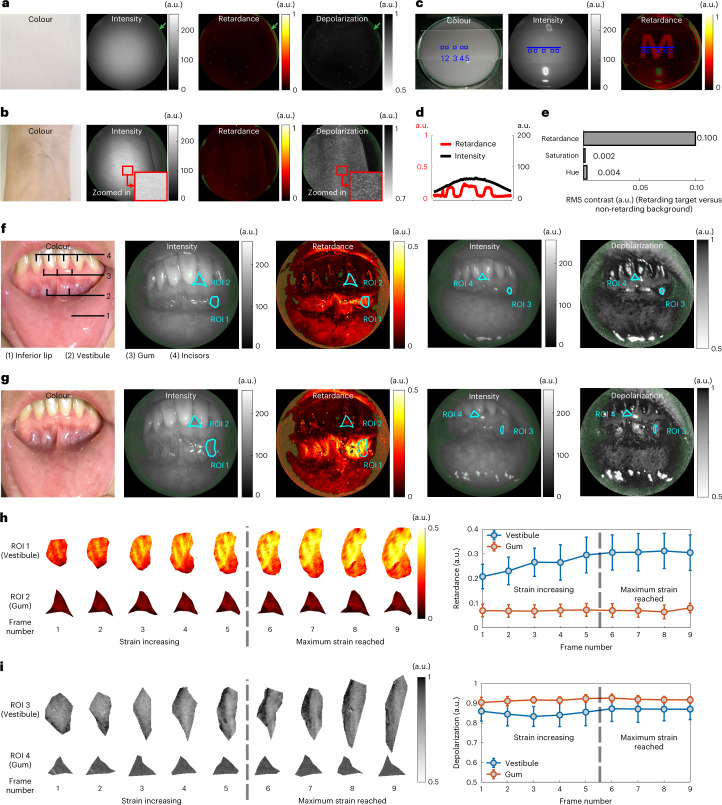
Validation of the SPE system. **a**, Imaging a homogeneous scattering target (white paper). The SPE revealed the expected high depolarization and low retardance. Regions rendered green (marked with green arrow indicators) indicate either under- or overexposure and were invalid. **b**, Imaging a volunteer’s skin, which was highly depolarizing and weakly retarding. Depolarization images show enhanced surface structure contrast compared with its intensity reference, visible in the enlarged regions. **c**, Imaging a phantom topped by an M-shaped retarding film. **d**, Retardance and intensity-reference profiles corresponding to the blue lines in **c**. **e**, RMS contrast in retardance, hue and saturation between the retarding target (squares 1, 3 and 5) and non-retarding background (squares 2 and 4) in **c**. **f**,**g**, Imaging a volunteer’s oral vestibule area without strain (**f**) and with strain (**g**). **h**, Change of retardance properties within the oral vestibule and the gum represented by ROI 1 and ROI 2 respectively in the lip stretching process recorded over nine consecutive frames. **i**, Change of depolarization properties within the oral vestibule and the gum represented by ROI 3 and ROI 4 respectively in the lip stretching process recorded over nine consecutive frames. Data in **h** and **i** are presented as mean ± standard deviation of the pixel values in the ROIs. Please refer to Supplementary Videos 1 and 2 for the entire process with full field of view. Source data

We validated the SPE system by imaging phantoms biological tissues with known polarization characteristics. White paper is a highly scattering homogeneous target with strong depolarization and minimal retardance^24^, as revealed by SPE (Fig. 2a). Similarly, skin is highly scattering with strong depolarization and weak retardance^33^, as demonstrated by imaging a volunteer’s arm skin in vivo with SPE (Fig. 2b). The skin surface structures were enhanced via SPE-depolarization imaging, consistent with previous studies^33,39,40^.

To benchmark SPE-retardance imaging, a tissue phantom with two layers was constructed (Methods). Its top layer consisted of an M-shaped retarding target to mimic retarding structures of biological tissues near surface. Its bottom layer was a non-retarding scattering medium. Although barely perceptible in the colour photo and the intensity-reference image, the target was distinguishable from background during SPE-retardance imaging (Fig. 2c). The intensity profile around the target was a smooth arc, while the retardance profile revealed sharp steps highlighting distinct retardance properties between the target and the background (Fig. 2d). The root-mean-square (RMS) contrast (Methods) between the target (squares 1, 3 and 5) and background (squares 2 and 4) was calculated for retardance and colour (hue and saturation) imaging, respectively. The retardance contrast revealed by SPE is approximately two orders of magnitude higher than that for conventional colour imaging (Fig. 2e). The target that has similar depolarization properties to the background showed a slightly higher depolarization value during SPE-depolarization imaging (Supplementary Fig. 3). This should stem from the equation used for depolarization image reconstruction (equation (4) in Methods), which under-estimates the degree of polarization of the light emerging from retarding objects, resulting in slight over-estimation of depolarization.

To assess the capability of SPE to work with a non-stationary target in vivo, we consecutively imaged a volunteer’s oral vestibule during manual stretch of the inferior lip (Supplementary Videos 1 and 2). Before the stress was applied (Fig. 2f), compared with colour imaging, SPE additionally revealed that the rich connective tissue of the vestibule was more retarding than the gum, and the gum was more depolarizing than the incisors, while the vestibule had a spatially varying depolarization. With the stress applied (Fig. 2g), SPE additionally detected (1) an increase in retardance for the stretched vestibule, which suggested better alignment of the connective tissue induced by the increasing strain^25,41,42^; (2) no variation in retardance for the gum, since the strain induced by stretching the lip did not transmit to the gum where no tissue deformation and displacement were observed; (3) depolarization of the oral vestibule and the gum essentially remained the same. SPE can monitor the continuous changes in polarization properties during this process due to its real-time capability. By tracking four representative regions across nine consecutive SPE frames (region of interest (ROI) 1, vestibule; ROI 2, gum under retardance mode; ROI 3, vestibule; ROI 4, gum under depolarization mode; for details, see Supplementary Fig. 4), SPE showed that the retardance in the vestibule continuously rose while the lip was being stretched, and then stabilized after the strain reached the maximum (stress could not further increase without causing discomfort), and also revealed that the retardance in the gum and the depolarization in both areas remained constant during the entire process (Fig. 2h,i). It is noted that the surface shape of the vestibule tissue analysed here was mainly determined by its rigid substrate below (bone and tooth root) that was locally flat and did not change very much in the stretching process (for details, see Supplementary Fig. 5). This experiment demonstrated the proof-of-concept of SPE imaging non-stationary target in vivo.

### Assessing the SPE in vivo in a laryngectomy case

To assess the potential role of SPE in laryngeal cancer surgery, we performed intra-operative in vivo imaging using SPE on a patient requiring routine total laryngectomy surgery to remove left hemi-larynx transglottic tumour (squamous cell carcinoma (SCC), proven via routine endoscopy and biopsy before the surgery). The surgical workflow was slightly altered for intra-operative SPE (see Methods about the ethics and workflow).

**Fig. 3:**
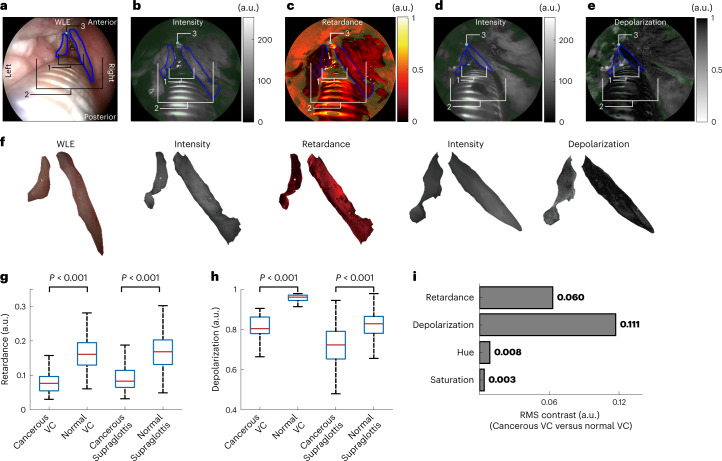
Assessment of SPE in vivo in a laryngectomy case. **a**, Larynx imaged with WLE: 1, glottis (vocal cords); 2, supraglottis; 3, anterior commissure. **b**,**c**, Retardance and its intensity-reference image of the larynx. **d**,**e**, Depolarization and its intensity-reference image of the larynx. **f**, Magnified images of the cancerous and normal vocal cord (for those of the supraglottis, see Supplementary Fig. 6). **g**–**h**, Retardance and depolarization values within the cancerous, normal vocal cord (VC) and the cancerous and normal supraglottis. In the box plots, the red centre line denotes the median value and the blue box shows the 25th and 75th percentiles of the dataset. The black whiskers mark the non-outlier minimum and non-outlier maximum. *P* values were calculated via a two-sided Mann–Whitney *U* test for two-group comparison. **i**, RMS contrast between the cancerous and normal vocal cords in terms of retardance and depolarization revealed by the SPE and hue and saturation shown in WLE. For consecutive SPE imaging of this case, please refer to Supplementary Videos 3 and 4. Source data

The WLE illustrated that the left hemi-supraglottic area showed loss of surface shape regularity and asymmetry to the right normal counterpart (Fig. 3a). The carcinoma in the left hemi-supraglottic area abutting the endotracheal tube was conspicuous due to either paler or darker red appearance than the light-pink normal tissue. However, the left vocal cord (proven cancerous post-operatively) did not show noticeable colour and texture contrast from the normal right vocal cord, with both presenting similar light-pink colour. This is a typical example where the colour/texture appearance manifested by the WLE results in difficulty to differentiate pathologies.

The SPE revealed that the lesions in the left hemi-supraglottic area presented loss of retardance compared with the right supraglottic area (Fig. 3b,c and Supplementary Video 3). The same appearance could also be observed clearly in the glottic areas: the left was less retarding than the right. The SPE also illustrated that the depolarization of the left cancerous supraglottis area was remarkably weaker than the normal right (Fig. 3d,e and Supplementary Video 4). The normal right vocal cord featured the highest depolarization in the larynx, in contrast to the cancerous left counterpart with moderate depolarization. The difference of the retardance and depolarization values for the cancerous lesions and the normal tissue in the larynx is statistically significant (*P* < 0.001, two-sided Mann–Whitney *U* test; for details, see Methods) (Fig. 3g,h). Note that some blood that is strongly absorbing re-appeared on the tissue surface during polarimetric endoscopy, resulting in underexposed regions in the SPE images. Strong specular reflections from smooth tissue surfaces also caused overexposed/pixel-saturated regions. These regions were rendered green in Fig. 3b–e and excluded from analysis. To elucidate the complementary contrast that can be provided by SPE, the glottic area was segmented and magnified in Fig. 3f. The RMS contrast between cancerous (left) and normal (right) vocal cords provided by SPE is approximately one order of magnitude higher than that presented by WLE (Fig. 3h). It was found that the polarimetric signatures of the normal larynx and cancerous tissue are different and the SPE may differentiate cancer not easily identifiable in WLE.

### Ex vivo polarimetric imaging and pathological study

**Fig. 4:**
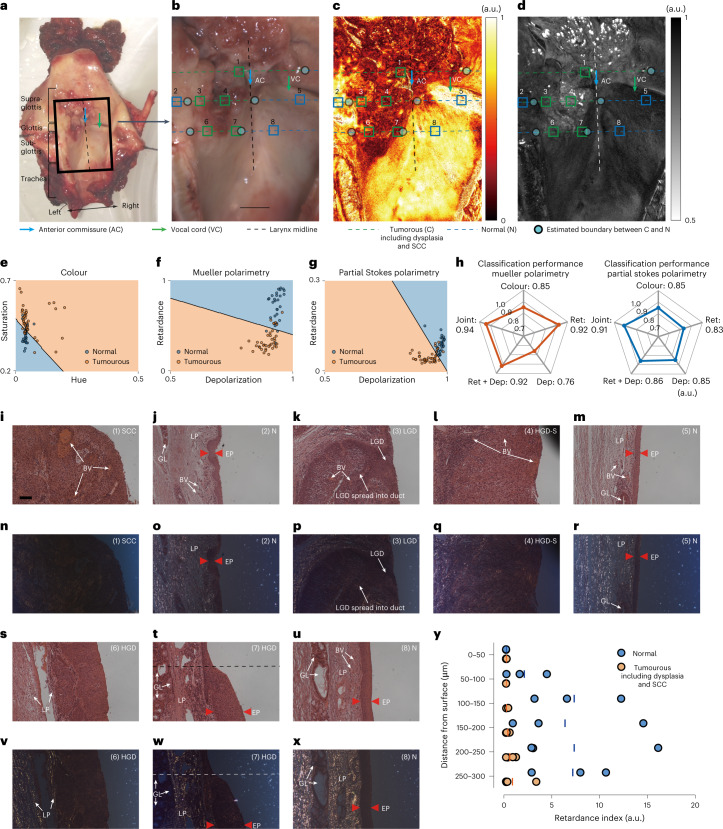
Ex vivo polarimetric imaging and pathological study. **a**, Photo of the excised larynx split in posterior midline and splayed open. The photo was taken immediately upon devascularization and before formalin fixation. **b**–**d**, White light, retardance and circular depolarization (obtained from Mueller polarimetry) images respectively of the area enclosed by the black box in **a**, labelled with diagnosis results from histopathology along three lines within supraglottis (**b**), glottis (**c**) and subglottis (**d**). Scale bar, 5 mm. **e**–**g**, Tissue pathology classification based on support vector machine using colour information, polarimetric information obtained from Mueller polarimetry and partial Stokes polarimetry respectively for 66 separate regions along the three lines in **b**–**d**. **h**, Classification performance measured by AUC of ROC curves (for all the ROC curves involved here, see Supplementary Figs. 8 and 9) using colour, retardance (Ret) and depolarization (Dep) information alone, using both retardance and depolarization (Ret + Dep) and using all of them (joint) altogether obtained from Mueller polarimetry (left) and partial Stokes polarimetry (right), respectively. **i**–**x**, White-light microscopy and polarization microscopy of the cross-sectional tissue slices of the larynx in ROIs 1–8 labelled in **b**–**d**. **i**–**x** have the same scale bar (100 μm) labelled in **i**. EP, epithelium; GL, glands; BV, blood vessels. ROI 1: SCC; ROIs 2, 5 and 8: normal represented by N; ROI 3: low-grade dysplasia spreading into the duct (LGD); ROI 4 is carcinoma with a thick high-grade dysplastic surface (HGD-S); ROI 6 is high-grade dysplasia (HGD); ROI 7 below the dash line is also high-grade dysplasia (HGD), above the dashed line is a transition area from dysplasia to normal. **y**, distribution of tissue retarders along the axial direction for ROI 1–8 in **i**–**x**. For normal tissue regions (ROIs 2, 5 and 8), the retardance indices (Methods) are small at 0–50 μm depth where epithelium locates and substantially increase from 100 μm below where LP locates. The retardance indices for cancerous regions including dysplastic ones (ROIs 1, 3, 4, 6 and 7) are constantly small across 0–300 μm depth. Source data

To validate the contrast observed during in vivo SPE imaging, we imaged the unfixed excised larynx (Fig. 4a) with a Mueller matrix polarimeter immediately after the laryngectomy surgery and before histopathology. The retardance and depolarization images were then reconstructed from the Mueller matrix image (Methods). The colour image labelled with results from histopathology (Fig. 4b) showed that most of the cancerous mass lesion demonstrated either paler or redder colour than the normal light-pink tissue. However, some light-pink regions were diagnosed as cancerous, for example ROI 3 within the left vocal cord area diagnosed as cancerous (low-grade dysplasia spreading down ducts, a type of pre-cancer) and ROIs 6 and 7 in the subglottis diagnosed as cancerous (high-grade dysplasia, a type of pre-cancer). The reconstructed retardance and depolarization images revealed good agreement with the in vivo results, namely that: (1) cancerous lesions in all the parts of larynx were of low retardance and low depolarization in general; (2) the normal vocal cord and subglottis on the right were strongly retarding and depolarizing, in contrast with the cancerous regions; (3) the normal vocal cord on the right featured the highest depolarization in the larynx (Fig. 4c,d). Those cancerous lesions that did not manifest abnormal colour like ROIs 3, 6 and 7 showed weaker retardance and lower depolarization in general than the normal, providing useful clues to detect the pathologies.

To assess the ability of white-light imaging and polarimetric imaging to differentiate cancerous lesions (including pre-cancer such as dysplasia and invasive cancer such as SCC) from normal tissue, binary linear classification of tissue pathologies based on a support vector machine was performed using colour, retardance and depolarization information (Fig. 4e–g) obtained from Mueller polarimetry and partial Stokes polarimetry as used for SPE in 66 independent regions within the supraglottis, glottis, and subglottis, respectively (Fig. 4b–d), with histopathology providing a standard (66 regions covered all the areas along the three lines in Fig. 4b–d where cross-sectional tissue slices were prepared for histopathology; for details, see Methods). The retardance and depolarization images reconstructed from partial Stokes polarimetry are shown in Supplementary Fig. 7. The area under the curve (AUC) values of receiver operating characteristic (ROC) curves indicate that classification performance based on Mueller polarimetry is mainly determined by retardance rather than depolarization; colour information can be beneficial to complement polarimetric imaging and improve the classification performance (Fig. 4h). For classification based on partial Stokes polarimetry, it was found that using retardance and depolarization together is better than using them individually; the best performance is achieved via combining both colour and polarimetric information. The classification based on partial Stokes polarimetry slightly underperformed that based on Mueller polarimetry, as expected. As a complement to standard-of-care WLE (sensitivity and specificity of 86% and 77%, respectively), the proposed polarimetry method may increase sensitivity and specificity values to 93% and 85%, respectively (for details, see Supplementary Figs. 8 and 9).

To investigate the origin of the retardance contrast demonstrated in polarimetric imaging, we used polarization microscopy to image the cross-sectional tissue slices of the larynx stained with haematoxylin and eosin (H&E) near ROIs 1–8 (Fig. 4i–x). Retarding compositions in tissue slices shows unique bright golden or dark-green colour under polarization microscopy, resulting in remarkable contrast^43,44^. The epithelium including lining of normal larynx surface as well as the duct of glands in the larynx was weakly retarding, revealed by polarization microscopy, while the lamina propria (LP)—a thick layer of connective tissue beneath the epithelium consisting of a well-structured network of collagen fibrils—manifested strong retardance (Fig. 4o,r,x). For normal larynx tissues, the epithelium was thin (50–100 μm thickness with about five to ten layers of cells) and smooth^45^. In polarimetric imaging, the epithelium lightly depolarizes the incident light, and a substantial portion remains circularly polarized when reaching the LP. The retarding LP converts the polarization state of this portion from circular to elliptical and linear during transit and scattering within the LP. The thin epithelium then lightly depolarizes the backpropagated light again before it emerges from the tissue surface. As a result, the emerging light contains a considerable component of linear polarization, which is the reason why normal larynx tissue presents high retardance in wide-field polarimetric imaging.

However, in the cancerous regions with invasive cancer, uncontrolled cancer cell proliferation disrupted normal tissue architectures by destroying the structure of the epithelium and invading the LP. Polarization microscopy revealed that the retarding fibrils within several hundred microns of the tissue surface were replaced by weakly retarding SCC (ROI 1, Fig. 4n) or SCC overlaid with a non-retarding high-grade dysplastic surface (ROI 4, Fig. 4q; the dysplastic surface was about 700 μm thick). In the regions with pre-cancer (ROIs 6 and 7, Fig. 4v,w), the weakly retarding epithelium thickened (thickness of epithelium is about 400 μm in ROI 6, and 250–300 μm in ROI 7, in comparison with 50–100 μm for ROIs 2, 5 and 8), resulting from abnormal proliferation of the epithelial cells. Although the retarding LP remains intact, it becomes located farther away from the tissue surface. Consequently, for the cancerous lesions (dysplasia and SCC both), there is a lack of retarding structures near tissue surface, in contrast to normal tissues (Fig. 4y).

The origin of depolarization contrast has been considered in previous studies on polarimetric imaging^20–22,46–49^ and polarized light scattering spectroscopy^50–52^ of scattering media. It was reported that cancerous human colon and cervix demonstrated weaker depolarization than normal^20,46,53,54^, consistent with the in vivo and ex vivo larynx results. Weaker depolarization mainly arises from increase of absorption due to enhanced vascularization of the tumour (enhanced vascularization near the tissue surface observed in Fig. 4i,k) and reduction in scattering due to the destruction of normal tissue architectures, as reported in refs. ^46,54–56^. Loss of retardance near the tissue surface also contributes to a reduction of depolarization in the cancerous regions.

The microscopy results agree well with the observations from wide-field polarimetric imaging that cancerous lesions consistently demonstrated lower retardance and weaker depolarization than normal tissues and suggest that the polarization contrast provided by SPE arises from the changes of tissue architectures that accompany the development of laryngeal cancer.

### Testing SPE during transoral surgery

**Fig. 5:**
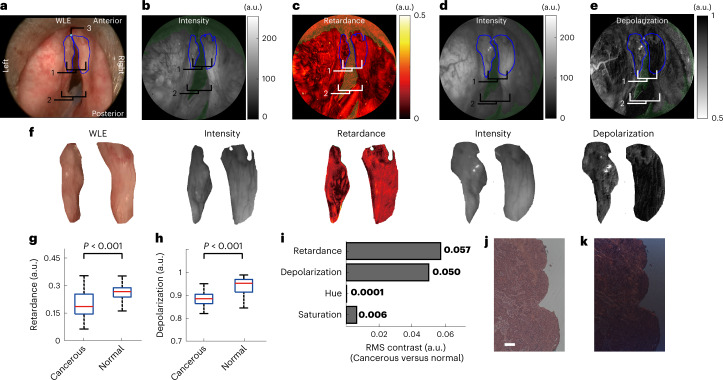
Testing SPE in vivo in a transoral surgery. **a**, Larynx imaged with WLE: 1, anterior vocal cords; 2, posterior vocal cords. **b**,**c**, Retardance (**b**) and its intensity-reference image (**c**) of the larynx. **d**,**e**, Depolarization (**d**) and its intensity-reference image (**e**) of the larynx. **f**, Magnified images of the cancerous and normal anterior vocal cords. **g**,**h**, Retardance (**g**) and depolarization (**h**) values within the cancerous and normal anterior vocal cord. In the box plots, the red centre line denotes the median value and the blue box shows the 25th and 75th percentiles of the dataset. The black whiskers mark the non-outlier minimum and non-outlier maximum. *P* values were calculated via a two-sided Mann–Whitney *U* test for two group comparison. **i**, RMS contrast between the cancerous and normal anterior vocal cords in terms of retardance and depolarization revealed by the SPE and hue and saturation shown in WLE. **j**,**k**, White-light microscopy (**j**) and polarization microscopy (**k**) of the tissue slices in the left anterior vocal cord diagnosed as cancerous (SCC). Scale bar, 100 μm. For consecutive SPE imaging of this case, please refer to Supplementary Videos 5 and 6. Source data

To further assess the reproducibility of SPE to differentiate pathologies based on contrast arising from tissue architectural changes, we further tested SPE on a patient requiring routine transoral laser surgery to remove a left glottic SCC lesion. The clinical workflow was adjusted as for the laryngectomy case, whereby SPE was tested after the routine anaesthesia for infraglottic jet ventilation and WLE inspection, and before routine transoral laser surgery. Although slight asymmetry can be observed with WLE (Fig. 5a), WLE did not reveal noticeable colour and texture contrast between the left anterior vocal cord (proven SCC by biopsy and surgical pathology) and the normal counterpart on the right. SPE revealed a suspect lesion in the left anterior vocal cord that was weakly retarding with a dark speckled appearance in the retardance image, compared to the right (Fig. 5b,c and Supplementary Video 4). This is in good agreement with the loss of retardance for cancer observed in the laryngectomy case. The anterior vocal cord on the left also presented a smaller depolarization, in comparison with the right vocal cord that was highly depolarizing (Fig. 5d,e and Supplementary Video 5). The anterior vocal cords under the different modalities have been segmented and magnified in Fig. 5f. The depolarization properties associated with normal and cancerous anterior vocal cord were fully consistent with the assessment for the laryngectomy case. The difference of the retardance and depolarization values for the cancerous and the normal anterior vocal cord is statistically significant (*P* < 0.001, two-sided Mann–Whitney *U* test) (Fig. 5g,h). The RMS contrast between the cancerous anterior vocal cord and the normal counterpart provided by SPE is much higher than that presented by WLE (Fig. 5i). Post-operative polarization microscopy confirmed the cancerous anterior vocal cord also had no LP near the tissue surface, associated with loss of retarding compositions (Fig. 5j,k), again consistent with the laryngectomy case. The posterior vocal cords showed good symmetry of shape, and similar colour, retardance and depolarization appearance by WLE and SPE, implying a consensus towards the normal status of the posterior vocal cords.

## Discussion

Surgical endoscopy plays an important role in minimally invasive procedures for laryngeal cancer. Surgeons rely on standard-of-care WLE because it provides a wealth of colour and surface information about lesions and the surgical scene. However, WLE is prone to limitations in differentiating those lesions without abnormal colour appearance or surface regularity, which may result in the inappropriate selection of biopsy areas, or in subtotal or unnecessary resection. Here we report a demonstration of SPE in a clinical setting by leveraging polarimetry to exploit tissue architectural information. We show how it may provide polarimetric contrast not available in WLE to better differentiate laryngeal cancerous and pre-cancerous lesions from normal tissue, and reveal the contrast arising from tissue architectural changes. The information from this additional endoscopic imaging modality complementing conventional WLE can enhance intra-operative laryngeal cancer detection. It may therefore confer the opportunity to ensure a more reliable assessment of tissue pathologies, clearer intra-operative guidance for biopsy and resection, and improved surgical care of laryngeal tumours. The SPE has unique advantages compared with other emerging endoscope techniques of high promise for cancer detection, such as endoscopic optical coherence tomography, fluorescence confocal endo-microscopy, non-linear endo-microscopy and scattering spectroscopy, which require additional ad hoc spatial scanning to form a wide-field image, and entail long acquisition times to achieve high definition or the acquisition of an image field that is different from the standard WLE view. The registration of the extracted information with conventional WLE and human vision is challenging but is required so that surgeons can percept and locate the lesions simultaneously. SPE has a wide field of view and high resolution, equivalent to conventional WLE and human vision, in addition to real-time performance. These features of SPE can ensure reasonably good image quality when dealing with surgical scenes that involve multiple moving and deforming objects where complex surgeon hand–eye coordination is essential. Furthermore, SPE can provide monochrome intensity-reference images that can facilitate the interpretation of the reconstructed polarimetric images. Moreover, the contrast given by SPE arises from the intrinsic mutation of tissue architectures during cancer pathogenesis. The label-free nature of the technique makes it simpler to integrate into existing surgical workflows and to be accepted from safety and regulatory points of view. Finally, SPE does not require costly light sources or components, with negligible additional cost compared with WLE (note that the cost of DoFP-LP image sensors is the same as normal image sensors under volume production).

We note the following limitations stemming from the fact that this present study provides proof-of-concept evidence for SPE. Firstly, we employed a simplified approach to reconstruct retardance and depolarization from partial Stokes polarimetry. This approach is pragmatic but not as rigorous as Mueller polarimetry. For example, highly retarding targets can be detected in the depolarization mode, as revealed in the phantom experiment. Since the useful information represented by the contrast between different tissue pathologies originally exists in the acquired partial Stokes polarimetric images, this simplified approach is viable to visualize that information in a more physically meaningful way. Referring to colour imaging with an RGB camera (or by naked eye), there are also overlaps among the red, green and blue colour channels in the wavelength domain while still being sufficient for real-world applications. Rigorously distinguishing the information from different channels is a ‘nice to have’ feature (rather than a ‘must have’ feature).

Secondly, there is a limit to the tissue depth through which light remains polarized, and it can be implied that SPE contrast mainly arises from the changes within a couple of hundred microns of the tissue surface. A comprehensive characterization of late-stage tumours and metastatic lesions located beyond this tissue depth is beyond the capability of SPE alone. Multimodal endoscopy integrating technology with deeper penetration depth but lower resolution, such as endoscopic ultrasound, may be necessary. However, we note that the superficial tissue volume is where epithelial cancers—including laryngeal tumours—are located at an early cancerous or pre-cancerous stage. Such lesions are prone to only very subtle colour changes, and it is therefore of importance to detect with a complementary imaging modality such as SPE.

Finally, like many biophotonics imaging techniques, tissue surface shape may affect the polarimetric values given by SPE. We assessed the impact of the shape on the polarimetric values acquired with SPE (Supplementary Figs. 10–12) and found that it does not affect recognition of the polarimetric contrast observed in the SPE images. In practice, the polarimetric values are not the only cue for tissue classification. For example, left–right anatomical structure symmetry (under WLE as well as SPE) is also important since cancerous areas may break symmetry and are straightforward to identify by surgeons. Cancers with no obvious shape change or asymmetry under WLE are important to address with SPE, and as a result, shape is not necessarily a variable that needs to be controlled for and will not prevent SPE from being used for tissue classification.

In this study, we enroled two patient volunteers to assess the SPE proof-of-concept. Although limited in number due to recruitment and time constraints, the laryngectomy cases were able to allow a thorough initial investigation and validation of the technique due to the different image fields and perspectives that could be captured. A larger cohort of patients would be possible by involving more medical centres, duplicating the SPE device, providing essential training regarding how to operate and sterilize/reuse the device and interpret the obtained polarimetric images, and enlisting the cooperation of surgical teams, pathologists and sterile service departments.

The SPE we described here entails a polarization-maintaining endoscope. Most of the commercially available rigid endoscopes are not polarization-maintaining normally, owing to the birefringent materials employed in the imaging channel. For example, rigid endoscopes from Karl Storz (a leading brand in the rigid endoscope market) have strong retardance varying across the field of view arising from the sapphire protective window at the distal end of the endoscope. In a larger-scale study and clinical translation this could be replaced by a non-birefringent material such as diamond so that polarization-maintaining rigid endoscopes can be reliably sourced. Another pathway is to perform complete snapshot Stokes polarimetric imaging instead of partial Stokes polarimetry with a typical non-polarization-maintaining endoscope. In theory, provided that the Mueller matrix image of the rigid endoscope is non-degenerate (and ideally with negligible depolarization and diattenuation) and invariant with working distance, its inverse could be post-multiplied by the Stokes measurements made at the proximal end to recover the polarization state at the distal tip. Our preliminary test confirmed that this proposal is also feasible.

Given that using colour and polarimetric information together can yield better performance, it would be of value to integrate these two modalities into one device. Such a device can be obtained by upgrading the current monochrome DoFP-LP image sensor to its colour version which has become commercially available recently as well as taking use of an endoscope light source with a removable narrowband filter (narrowband light for polarimetric imaging and broadband light for white-light imaging). The switch between SPE and WLE can be achieved by simply controlling the narrowband filter in the light source.

In summary, we have shown that SPE is a real-time, label-free, contact-free, wide-field, high-definition endoscopic imaging technique that can aid the intra-operative detection of laryngeal cancer. Our study also shows the translational potential and practicality of SPE during surgical imaging in vivo. We believe that SPE holds promise as an effective complementary method to standard-of-care WLE. The combination of SPE and routine WLE could open up opportunities for improving the surgical care of laryngeal tumours.

## Methods

### SPE system

The entire SPE system consists of five modules:

1. Rigid endoscope. A European Conformity (CE) approved commercial rigid endoscope with inbuilt imaging and illumination channels (COMEG, 6 mm diameter, 0° view angle) was used as the main imaging device. The 4-mm-diameter imaging channel is polarization maintaining, while the illumination channel uses a fully depolarizing fibre optic bundle that is distributed around a peripheral ring-shaped pattern at the tip.
2. Endoscopic polarizing tip attachment. This held a ring-shaped circularly polarizing plate over the illumination channel at the distal end of the endoscope to polarize the illumination light. The Stokes parameters of the illumination light were 1.000, −0.002, 0.028 and 0.999 characterized by a calibrated Stokes polarimeter. The disposable mount (8 mm outer diameter, 1 cm long) was 3D printed, as shown in Fig. 1d. It can be miniaturized if using traditional machine tools and materials of high strength like metal to reduce the diameter and the protruding design. Alternatively, an endoscope sheath can be made to mount the polarizing plate inside the distal tip of the sheath.
3. Polarization state analysing imager. The imager consisted of a DoFP-LP camera (PolarCam, 4D Technology) and a removable quarter waveplate (WP140HE, Edmund Optics) in a quick-release mount (QRC1A, Thorlabs). The DoFP-LP camera was mounted on an adjustable rotation stage with locking mechanism (CRM1/M, Thorlabs), so the angle between the DoFP-LP camera and the waveplate could be adjusted to 45° (parallel to the top right quadrant of each super-pixel in the DoFP-LP camera). A 50 mm focal length lens served as the objective for the imager. Its position was adjustable and lockable between the polarimetric camera and the eyepiece of the rigid endoscope for focusing.
4. Endoscope light source. A lamp (Lumen Pro 200, Prior Scientific) and a narrowband filter (FF01-543/22-25, Semrock) was used to provide narrowband illumination for the endoscope. The light was delivered to the endoscope light port via a liquid light guide. The wavelength spectrum emitting from the SPE was centred at 546 nm, as characterized by a spectrometer (HR4000-UV-NIR, Ocean Optics; Supplementary Fig. 2). The output radiant flux of the SPE was 30 mW characterized by a power meter (FieldMaxII, Coherent).
5. Computer and software. A custom-written LabVIEW program executed on a laptop computer (Latitude 7480, Dell) was used to run the SPE system, to read out and save the data, to apply the polarimetric correction of the output data, and to reconstruct and display retardance and depolarization and their intensity references.
6. Modules 1 and 2 were assembled securely before the surgery and sent to a third-party company for sterilization (process compliant with ISO13485). Module 3 was tethered to modules 4 and 5 by a liquid light guide cable and an ethernet cable before the surgery. When the surgical team were ready to image the patient, modules 1 and 2 were unpacked in the operating theatre from sterile storage and rapidly integrated with module 3. Note that all the modules except module 2 were reused.

### Image processing and reconstruction

The output data from SPE were processed as follows:

1. Polarimetric correction: DoFP-LP cameras incorporate an array of micro-linear-polarizers consisting of an aluminium nano-wire grid aligned at 90°, 45°, 135° and 0° on the top of the photodiode array. The fixed pattern noise arising from variable diattenuation of the micro-linear polarizers was calibrated according to ref. ^57^. The calibration result is camera specific but is normally performed only once for a particular DoFP-LP camera.
2. De-mosaicing: We used bilinear interpolation to de-mosaic the polarimetric corrected image data and generated four 1,384 × 1,208-pixel subimages *I*_90_, *I*_45_, *I*_135_ and *I*_0_, respectively.
3. Reconstruction of partial Stokes polarimetric images:
  For the retardance mode of SPE, the quarter waveplate in module 3 was removed to obtain the first three elements of Stokes parameters *S*_0_, *S*_1_ and *S*_2_,
  1 $$\begin{array}{l}S_0 = \left( {I_0 + I_{45} + I_{90} + I_{135}} \right)/2\\ S_1 = I_0 - I_{90}\\ S_2 = I_{45} - I_{135}\end{array}$$
  For the SPE depolarization mode the 45° quarter waveplate was placed in module 3 and the subimages *I*_0_ and *I*_90_ effectively correspond to the left and right circularly polarized light images. The first and fourth elements of Stokes parameters *S*_0_ and *S*_3_ were obtained,
  2 $$\begin{array}{l}S_0 = I_0 + I_{90}\\ S_3 = I_0 - I_{90}\end{array}$$
4. Reconstruction of retardance, depolarization and their intensity references:
  The reconstruction method was based on ref. ^33^. Tissue Mueller polarimetric studies indicate that depolarization and linear retardance are found to be the main polarization characteristics of interest and utility, and the magnitude of diattenuation for the majority of tissue types is typically very small^58,59^, with only a small number of exceptions such as skeletal and heart muscles^60^. For tissues with very low diattenuation like the larynx, the main mechanism to convert circular to linear polarized light is tissue retardance^33–36,61^. Given that fully circularly polarized light is used for illumination, the magnitude of retardance *A*_*δ*_ is therefore determined by the linearly polarized components within the emergent light, presented by the degree of linear polarization for the emergent light,
  3 $$A_\delta = \left| {\sin \delta _{\mathrm{L}}} \right| = \frac{{\sqrt {S_1^2 + S_2^2} }}{{S_0}}$$
  where *δ*_**L**_ is linear phase retardance (in radian), and *S*_0_, *S*_1_ and *S*_2_ are obtained from equation (1). *A*_*δ*_ is a dimensionless quantity with minimum value 0 (non-retarding) and maximum value 1 (highly retarding), and was used to reconstruct the retardance images. The *S*_0_ image generated from equation (1) serves as the intensity-reference image for the retardance mode of the SPE.
  The circularly polarized illuminating light can maintain its polarization after one or small number of scattering events, while multiple scattering randomizes the polarization. The magnitude of depolarization *A*_dep_ can thus be characterized by the proportion of the randomly polarized backscattered light, as follows
  4 $$A_{{\mathrm{dep}}} = \frac{{{\mathrm{RP}}}}{T} = 1 - \frac{{{\mathrm{PM}}}}{T} = 1 - \left| {S_3/S_0} \right|$$
  where RP, PM and *T* refer to the randomly polarized, polarization-maintaining and total backscattered light intensity, respectively. Equation (4) is effectively the complement of the degree of circular polarization for the emergent light. Note that the total and the polarization maintaining intensity are given by the first and fourth elements of the Stokes parameters *S*_0_ and *S*_3_ obtained from equation (2), respectively. *A*_dep_ is a dimensionless quantity with minimum value 0 (non-depolarizing) and maximum value 1 (fully depolarizing), and was used to reconstruct the depolarization images. The *S*_0_ image generated from equation (2) is the intensity-reference image for the depolarization mode of the SPE. As A_dep_ may overestimate depolarization for strongly retarding turbid media, since the emergent light may contain linearly polarized components, RP may be slightly smaller than *T* − PM in equation (4).
  It is noted that the retardance and depolarization obtained from this simplified approach based on partial Stokes polarimetry used for SPE is not as rigorous as that based on Mueller polarimetry.
5. Optional processing of intensity-reference image:

The intensity-reference images can be dark, especially for the areas at the periphery of the field of view due to inhomogeneous endoscope illumination, slight vignetting and presence of strongly absorbing objects such as blood in addition to generally lower illumination power of the SPE system compared with standard endoscopy. An optional gamma correction could be applied to the intensity-reference image according to equation (5) to improve the visibility of these regions.

5 $$S_{0\_{\mathrm{out}}} = \left( {S_{0\_{\mathrm{in}}}} \right)^{{\mathrm{gamma}}}$$

S_0_in_ and S_0_out_ are the intensity reference before and after the gamma correction. A gamma value of 1/2 was used for the results presented in this work. To address degradation of image contrast caused by gamma correction, the intensity-reference images were enhanced with a built-in image sharpening function (imsharpen) in MATLAB. Additionally, underexposed (caused by, for example, blood on the tissue surface) and overexposed (caused by, for example, specular highlights) areas, referred to as low-image-quality areas, were detected by thresholding the intensity-reference image before gamma correction,

6 $$\begin{array}{l}{\mathrm{Overexposed}}:\frac{{S_{0\_{\mathrm{in}}}}}{2} > 95{{{\mathrm{\% }}}} \times \left( {2^{{\mathrm{bitdepth}}} - 1} \right)\\ {\mathrm{Underexposed}}:\frac{{S_{0\_{\mathrm{in}}}}}{2} < 3{{{\mathrm{\% }}}} \times \left( {2^{{\mathrm{bitdepth}}} - 1} \right)\end{array}$$

where bitdepth refers to the bit depth of the raw output images from the LP DoFP camera, which was 8 in this work. Low-image-quality areas may not be imaged by the SPE system reliably and were therefore rendered green to avoid misleading viewers. Note that optional processing of the intensity-reference image was not executed during intra-operative SPE imaging.

### Preparation of the tissue phantom

The two-layer tissue phantom was constructed to assess the SPE imaging ability. The bottom layer was prepared in a glass Petri dish from 1 ml 10% intralipid and 0.01 ml India ink dissolved in 15 ml distilled water to simulate tissue scattering and absorption at 546 nm, respectively. The scattering, absorption and polarization property for this layer can found in refs. ^62,63^ and ref. ^25^, respectively. The top layer consisted of an ‘M’-shaped piece of transparent birefringent film to simulate retarding structures near tissue surface. The retardance of the birefringent film used was spatially uniform, confirmed by examination with a pair of orthogonal linear polarizers.

### RMS contrast

A common way to define the image contrast in an image is to measure the RMS contrast^64^, defined as

$${\mathrm{contrast}} = \sqrt {\frac{1}{{n - 1}}\mathop {\sum}\limits_{i = 1}^n {\left( {x_i - \overline x } \right)^2} }$$

where

7 $$\overline x = \frac{1}{n}\mathop {\sum}\limits_{i = 1}^n {x_i} ,\,{{{\mathrm{and}}}}\,0 \le x_i \le 1$$

where *n* and *x*_*i*_ are the pixel number and an individual pixel value of the image normalized to the range from 0 to 1. We extended the use of the RMS contrast to compare the median values (of retardance, depolarization, hue or saturation) between the two categories (that is, cancerous versus normal) as follows:

$${\mathrm{contrast}} = \sqrt {\mathop {\sum}\limits_{i = 1}^2 {\left( {m_i - \overline m } \right)^2} } ,$$

where

8 $$\overline m = \frac{1}{2}\mathop {\sum}\limits_{i = 1}^2 {\overline m _i} \,{{{\mathrm{and}}}}\,0 \le m_i \le 1$$

where *m*_*i*_ is the median value of each category. Note that the ranges of the retardance and depolarization reconstructed according to equations (3) and (4), and the hue and saturation converted with the MATLAB built-in function (‘rgb2hsv’), are all from 0 to 1, and did not require additional normalization.

### Surgical workflow for the laryngectomy case

The surgical workflow was slightly altered for intra-operative SPE, consisting in sequence: (1) routine general anaesthesia with endotracheal tube intubation; (2) routine WLE to inspect tumour bulk in the larynx and to remove secretions and blood obstructing the view, during which time the sterile part of the SPE was unpacked and integrated; (3) transorally introducing surgeon-controlled SPE and advancing to the larynx in depolarization mode (about 1 min); (4) removal of SPE to switch mode, re-introduction and surgeon-controlled SPE in retardance mode (1 min); (5) routine laryngectomy where the SPE was not involved; (6) ex vivo imaging of the freshly resected larynx tissue immediately after laryngectomy with a benchtop Mueller polarimetric imaging system in theatre; (7) routine completion of the surgery. The tissue slides of the resected larynx were collected post-operatively for further standard clinical and additional histopathological validation.

### Quantitative analysis of the SPE images and statistics

Quantitative analysis of the reconstructed retardance and depolarization images acquired during the in vivo study was performed for comparison of polarization properties of laryngeal tissues in Figs. 3 and 5. To avoid the interference from the high-frequency texture and noise, all the analysed images—including retardance, depolarization and colour—were divided into a grid of unit size 16 × 16 pixels (16 pixels roughly correspond to 0.5–0.7 mm). The mean values of these units were treated as statistically independent and used for descriptive and inferential analysis. Based on segmentation of different anatomical parts of larynx with underexposed and overexposed areas excluded, for the laryngectomy case, there are 74, 240, 324 and 646 separate unit regions for the left vocal cord, the right vocal cord, the left supraglottis and the right supraglottis, respectively, extracted for analysis of the SPE-retardance image, and 38, 77, 265 and 1,835 separate unit regions, respectively, for analysis of the depolarization image; for the transoral surgery case, there are 87 and 141 separate unit regions for the left and right upper vocal cord, respectively, for analysis of the retardance image, and 147 and 190 separate unit regions for the depolarization images. The different tissue regions were classified by a professional pathologist and the otorhinolaryngologist who performed the surgery post-operatively on the basis of identification of the anatomical landmarks and histopathology.

### Mueller polarimetry for the ex vivo study and histopathology

A separate benchtop Mueller polarimeter was set up in reflection mode to investigate the polarization properties of freshly excised laryngeal tissues. The polarimeter consisted of a light source, a polarization state generator (PSG), a polarization state analyser and a camera. The light source was the same (Lumen200Pro, Prior Scientific) with SPE. The PSG constituted a 0° linear polarizer and a quarter waveplate mounted in a motorized rotation stage (PRM1/MZ8, Thorlabs) rotating to −45°, 0°, 30° and 60°. The PSA was a motorized rapid switching filter wheel (FW103H/M, Thorlabs) containing four linear polarizers with extinction ratio 9,000:1 (XP42-200, Edmund Optics) orientated at −45°, 0°, 45° and 90°, respectively and two circular polarizers (CP42HE, Edmund Optics) with one left polarized and the other right polarized. The image sensor was a CCD camera (Blackfly, FLIR). The acquisition time the system is typically about 15 s per Mueller polarimetric image. The polarimeter was calibrated on the basis of the eigenvalue calibration method^65^ to obtain the real instrumental matrices of the PSG and PSA, which normally deviate from their nominal values. The typical mean and maximum elemental errors of the polarimeter were 0.35% and 1.3%.

Retardance and decomposition images were reconstructed from the Mueller matrix images according to the extended Lu–Chipman decomposition method^66–69^. The Mueller matrices were decomposed into depolarization, retardance and diattenuation matrices represented by *M*_∆_, *M*_R_ and *M*_D_. Total depolarization is given by ref. ^67^,

9 $$\begin{array}{l}T_{{\mathrm{dep}}} = 1 - \frac{{tr\left( {M_{\Delta}} \right) - 1}}{3}\\ A_{{\mathrm{dep}}} = 1 - \left| {M_{\Delta}\left( {4,4} \right)} \right|\end{array}$$

Since SPE depolarization corresponds circular depolarization, the magnitude of depolarization used to reconstruct the Mueller-depolarization image in Fig.  4d was *A*_dep_ determined by *M*_∆_(4,4), following the convention in refs. ^68,70^. The depolarization image of the rigid endoscope in Fig. 1e was reconstructed with *T*_dep_. The magnitude of retardance can be obtained^67^,

10 $$\begin{array}{l}R = \cos ^{ - 1}\left( {\frac{{tr\left( {M_{\mathrm{R}}} \right)}}{2} - 1} \right)\\ A_\delta = \sin R\end{array}$$

where *A*_*δ*_ was used to reconstruct the retardance image shown in Fig. 4.

After being imaged by the Mueller polarimeter, the resected specimen was sent for histopathology where it was fixed for 24 h and then transversely sliced with a bandsaw at a separation of 5 mm, from superior to inferior. The bandsaw-sliced samples were decalcified using 10% formic acid, processed with paraffin, embedded onto wax blocks, cut into 6 μm sections using microtome and stained with H&E. The correlation along the superior–inferior laryngeal axis, between the Mueller polarimetric images and the histological slices, was made on the basis of the sequence of the bandsaw-sliced samples, as well as identification of the anatomical landmarks such as vocal cord, anterior commissure and so on, and unique tissue features such as bumps. Note that only the top or the bottom part of the bandsawed slices were cut with microtome. The correlation along the left–right direction for the near-glottic regions used for analysis in this work was based on the distance laterally outwards from the anterior midline of the larynx. The diagnosis of the tissue regions was made by a professional pathologist, and the regions were classified by the professional pathologist together with the otorhinolaryngologist who performed the surgery. The World Health Organization classification of head and neck tumours was referred to for the diagnosis^71^.

### Classification based on linear SVM

The areas along the three lines in Fig. 4b–d that correspond to the three tissue histological slices were analysed. The areas along each line were divided into a row of 30 × 30-pixel independent unit regions positioned next to each other, resulting in 22 samples for each line. The mean retardance, depolarization, hue and saturation of these units and the corresponding histopathological diagnostic information (SCC and dysplasia versus normal) were extracted. Sixty-six samples were obtained totally and randomly split into training and testing datasets in a ratio of 0.6 over 0.4. A binary classifier based on a support vector machine with a linear kernel was trained with the training set, with the ROC curve and the AUC value computed with the test set for comparison. These were implemented in Python 3.7.4 with scikit-learn 0.21.3, matplotlib 3.1.1 and mlxtend 0.17.2.

### Polarization microscopy and polarization image analysis

Polarization microscopy was conducted on an inverted transmission microscope (IX71, Olympus) supporting polarization and white-light microscopy modes. A built-in halogen lamp, a 10× microscope objective and a colour camera (Blackfly, FLIR) acquired the images in Fig. 4. The preliminary tests in ref. ^43^ confirmed no substantial change in dichroism/birefringence of H&E-stained tissue compared with unstained samples. We used H&E staining for pathology. The system first ran in white-light microscopy mode to locate the region to be imaged and to adjust the acquisition settings. Then, the system was switched to polarization microscopy mode by inserting an orthogonal pair of linear polarizers in the illumination and imaging channels, respectively. The camera settings remained unchanged except that the exposure time was multiplied by 2,500. A polarization microscopy image was then captured. By thresholding in the hue domain of the captured polarization microscopy image, the retarding pixels showing golden or dark-green colour (encoded hue values ranging from 0.05 to 0.45 by MATLAB) were differentiated from non-retarding ones (Supplementary Fig. 16). The retardance index for an ROI was defined as the mean grey levels of the retarding pixels in that region as follows:

11 $${{{\mathrm{Retarding}}}}\,{{{\mathrm{Index}}}} = \left\{ {\begin{array}{*{20}{c}} {\frac{1}{n}\mathop {\sum}\limits_{n \in {\mathrm{RETARDING}}}^i {\left[ {0.2989,\,0.5870,\,0.1140} \right]} \left[ {\begin{array}{*{20}{c}} {r_i} \\ {g_i} \\ {b_i} \end{array}} \right],} & {{{{\mathrm{if}}}}\,n \ne 0} \\ {0,} & {{{{\mathrm{if}}}}\,n = 0} \end{array}} \right\}$$

where *n* is the number of retarding pixels in an ROI. Note that the grey level of a retarding pixel is converted from a weighted sum of the red, green and blue pixel values of that pixel with built-in MATLAB function ‘rgb2gray’.

### Colour-image acquisition

The reference colour images involved in vivo imaging of the patients, and Figs. 3a and 5a were taken from the preceding WLE. In Supplementary Information (Supplementary Section 10 and Supplementary Figs. 13–15) we also explored the feasibility to convert the intensity-reference generated by SPE into a virtual, fully correspondent WLE image via deep learning, although the virtual WLE images were not used for analysis. The colour images shown in Figs. 2a–c,e,f and 4a were taken with a mobile phone camera. The colour image in Fig. 4b was obtained with the colour camera used in the imaging Mueller polarimeter setup (only the green channel of the camera was used for the Mueller polarimetric study). Using the same camera for polarimetric and colour images ensured a full correspondence between different imaging modes.

### Human research participants

The participants involved in the study of oral-cavity imaging, the laryngectomy case and the transoral surgery case were a 32-year-old male, a 64-year-old female and a 63-year-old male, respectively. Informed consent was obtained from all the participants. None of the participants received compensation. Ethical approval was obtained from NHS Central London Research Ethics Committees (reference number 10/H0718/55) and the Joint Research Compliance Office Imperial College London (reference number 20IC5863).

### Reporting summary

Further information on research design is available in the Nature Portfolio Reporting Summary linked to this article.

## Supplementary information

Supplementary Information Supplementary discussion, figures, video captions and references.

Reporting Summary

Supplementary Video 1 SPE-retardance imaging of the volunteer’s oral vestibule.

Supplementary Video 2 SPE-depolarization imaging of the volunteer’s oral vestibule.

Supplementary Video 3 SPE-retardance imaging of the patient requiring laryngectomy.

Supplementary Video 4 SPE-depolarization imaging of the patient requiring laryngectomy.

Supplementary Video 5 SPE-retardance imaging of the patient requiring transoral surgery.

Supplementary Video 6 SPE-depolarization imaging of the patient requiring transoral surgery.

## Data Availability

The data supporting the results in this study are available within the paper and its Supplementary Information. Source data are provided with this paper. The raw images involving human participants are protected owing to data-privacy requirements, and can be made available for research purposes on reasonable request from the corresponding authors, provided that approval is obtained after an institutional review procedure at Imperial College London and London North West University Healthcare NHS Trust. The response from the authors will usually be within 4 weeks.

## Source data

Source data for Fig. 1 Source data.
Source data for Fig. 2 Source data.
Source data for Fig. 3 Source data.
Source data for Fig. 4 Source data.
Source data for Fig. 5 Source data.

## Code availability

The custom code used in this study is available on the Zenodo repository at 10.5281/zenodo.7435189.
