# Supplementary material for: Surgical polarimetric endoscopy for the detection of laryngeal cancer
Source: Nat Biomed Eng. 2023 Apr 3;7(8):971–85. doi: 10.1038/s41551-023-01018-0 (PMC10427430; doi:10.1038/s41551-023-01018-0)
Supplement: Supplementary file 1 — Supplementary discussion, figures, video captions and references. [file 41551_2023_1018_MOESM1_ESM.pdf]

nature biomedical engineering

Article

https://doi.org/10.1038/s41551-023-01018-0

Surgical polarimetric endoscopy for the detection of laryngeal cancer

In the format provided by the authors and unedited

Contents

1. Mueller matrices of the rigid endoscope used in the SPE. 2
2. Wavelength spectrum of the illumination light of the SPE system 3
3. Depolarization image of the letter phantom obtained with SPE 3
4. ROI tracking in the oral vestibule imaging experiment 4
5. Deformation of vestibule tissue that is stretched 4
6. Supraglottic regions in the *in vivo* experiment involving the patient requiring laryngectomy 5
7. Retardance and depolarization images of the *ex vivo* tissue reconstructed from partial Stokes polarimetry 5
8. Receiver operating characteristic (ROC) curves involved in the *ex vivo* tissue experiment 6
9. Evaluating the influence of the perspective viewing angle and tissue surface shape 7
10. Virtual white light endoscopy (WLE) with SPE via deep learning 10
11. Polarization image regions for retardance index calculation. 13
12. Using SPE to differentiate cancerous from inflammatory lesions: feasibility analysis 14
13. Supplementary video information 15
14. References 16

1. Mueller matrices of the rigid endoscope used in the SPE.

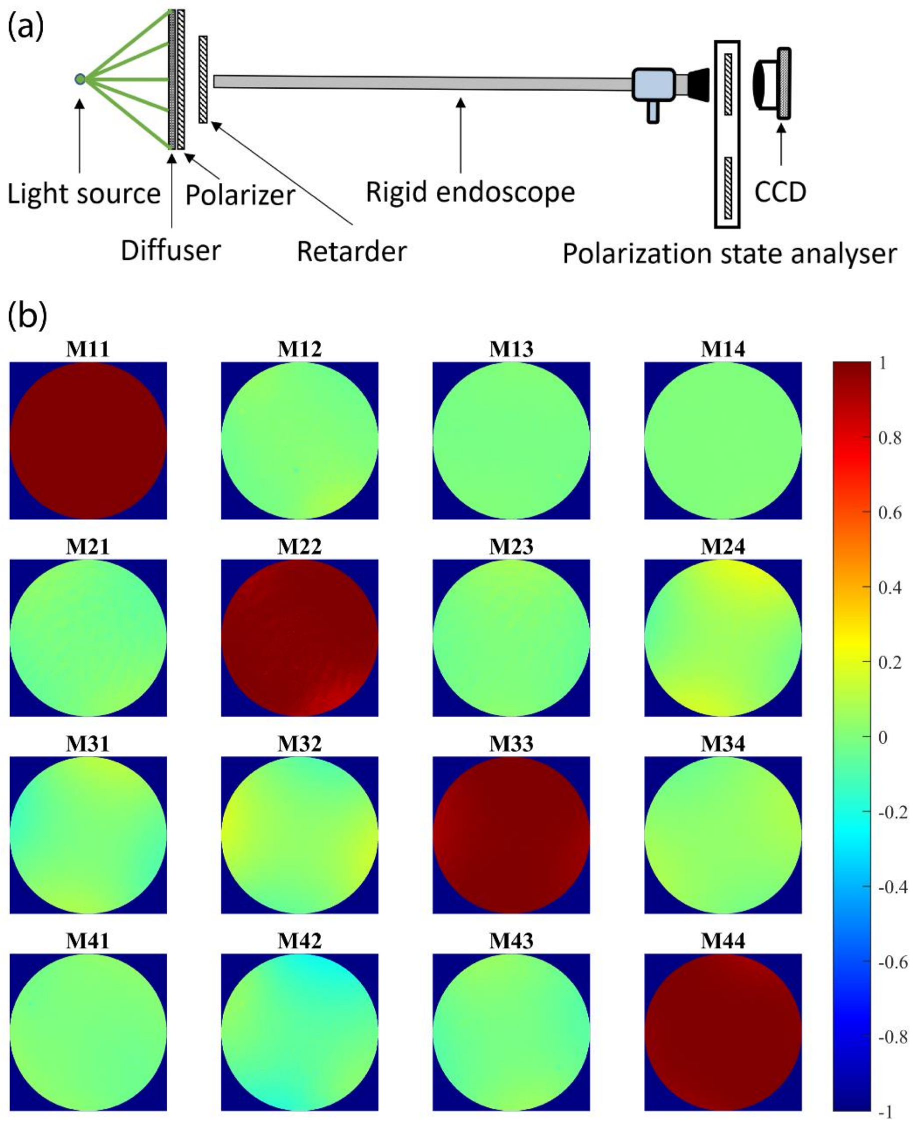

Figure S1. a. optical setup of the imaging Mueller polarimeter for measuring the imaging channel of the rigid endoscope used in the SPE system. b. Mueller matrix image of the imaging channel of the rigid endoscope.

An imaging Mueller polarimeter in transmission mode suitable for measuring the optical imaging devices with large angle of view was set up to investigate the polarization properties of the imaging channel of the rigid endoscope used in the SPE. The polarimeter consisted of a light source, a diffuser, a polarization state generator (PSG) with a large aperture, a polarization state analyzer (PSA) and a camera. The light source was the same to the one used in the major study, that is, a high-pressure mercury lamp (Lumen200Pro, Prior Scientific) with a band pass narrow band filter (FF01-543/22-25, Semrock). The PSG was comprised of a 0° linear polarizer mounted in a rotation mount with large clear aperture (RSP2D, Thorlabs) and a quarter waveplate mounted in a motorized rotation stage (PRM1/MZ8, Thorlabs) rotating to -45°, 0°, 30°, and 60°. The PSA was the same to that in the benchtop Mueller polarimeter in reflectance mode used in the major study, which was a motorized rapid switching filter

wheel (FW103H/M, Thorlabs) containing four linear polarizers orientated at −45° 0° 45° 90° and two circular polarizers with one left polarized and the other right polarized. A cooled CCD camera (Retiga Exi, Qimaging) was used to capture the radiometric images. The polarimeter was calibrated based on the eigenvalue calibration method to obtain the real instrumental matrices of the PSG and a PSA (1). During the measurement, the distance between the tip of the rigid endoscope and the waveplate in the PSG was minimized to fit the large angle of view of the rigid endoscope. The obtained Mueller matrices are highly diagonal, which suggests that the imaging channel of the rigid endoscope is highly polarization maintaining, namely, with negligible magnitude of depolarization, retardance and diattenuation. Lu-Chipman decomposition (2) was then used to quantify its depolarization, retardance and diattenuation properties that were shown in Figure 1(e) in the main text.

2. Wavelength spectrum of the illumination light of the SPE system

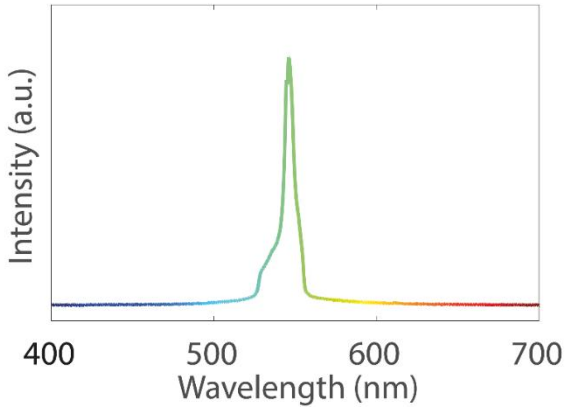

Figure S2. Wavelength spectrum of the illumination light of the SPE system measured with a spectrometer (HR2000, Ocean Optics). The spectrum was mainly determined by the emission spectrum of Mercury (with a peak at 546 nm) and the high thermal damage threshold narrow band filter centred at 543 nm with 22nm bandwidth we integrated into the light source. It was also affected by the transmission properties of the liquid light guide and the illumination channel of the rigid endoscope. The narrow band filter was used to remove UV radiation from the Mercury lamp and to reduce the potential spectral errors in the polarimetric systems.

3. Depolarization image of the letter phantom obtained with SPE

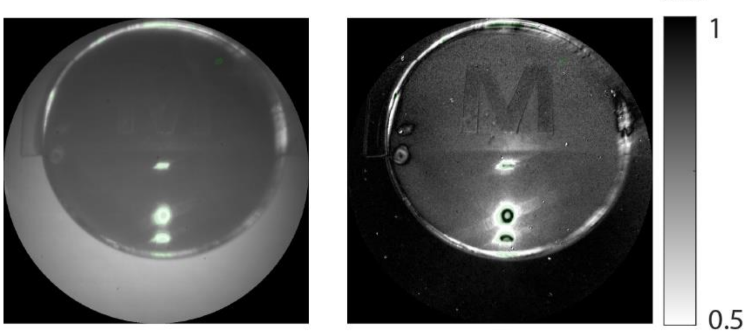

Figure S3. Left: intensity reference image; Right: depolarization image of the retarding phantom.

4. ROI tracking in the oral vestibule imaging experiment

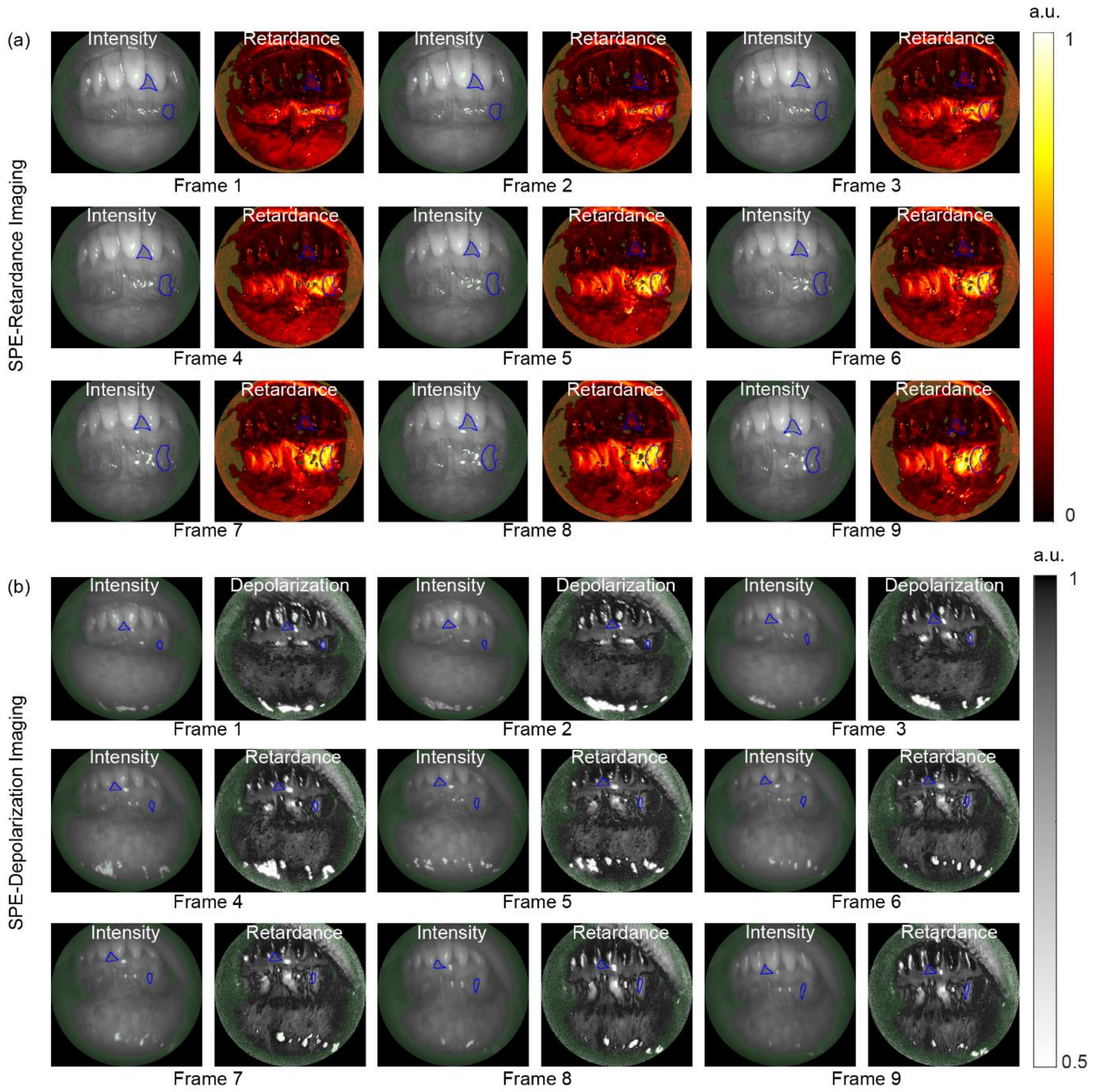

Figure S4. The ROI information in the oral vestibule imaging experiment demonstrated in the main text. Only two frames for each imaging mode were shown in the main text. Here the ROIs across all the nine frames for each mode were outlined in blue. (a) ROIs 1 and 2 within the oral vestibule and the gum respectively (retardance images), and (b) ROIs 3 and 4 within the oral vestibule and the gum respectively (depolarization images). The ROIs were segmented and tracked manually across nine consecutive frames using features (e.g. blood vessel patterns, edges) presented in their corresponding reference intensity images.

5. Deformation of vestibule tissue that is stretched

In the oral vestibule imaging experiment, we mainly imaged the gum and the oral vestibule below the gum and analysed how they changed when the lip was being stretched. The analysed vestibule area is indicated with a blue box in Figure S5 (right). In this area, the superficial soft tissue (epithelium, lamina propria etc.) attaches to rigid substrate (bone, tooth roots), and the surface shape in this area is mainly determined by the shape of the substrate that is locally flat. When stress was induced, the superficial

soft tissue became thinner and there was a small displacement between the soft tissue and the substrate. The surface shape, however, was still determined by the flat substrate and did not change very much (see Figure S5 left).

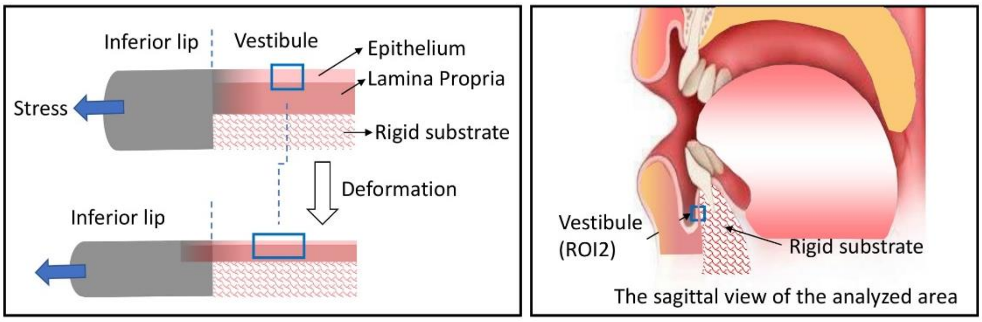

Figure S5. Left, deformation of tissue that is stretched. Right, the sagittal view of the analysed vestibule area (represented by ROI2 in the main manuscript).

6. Supraglottic regions in the *in vivo* experiment involving the patient requiring laryngectomy

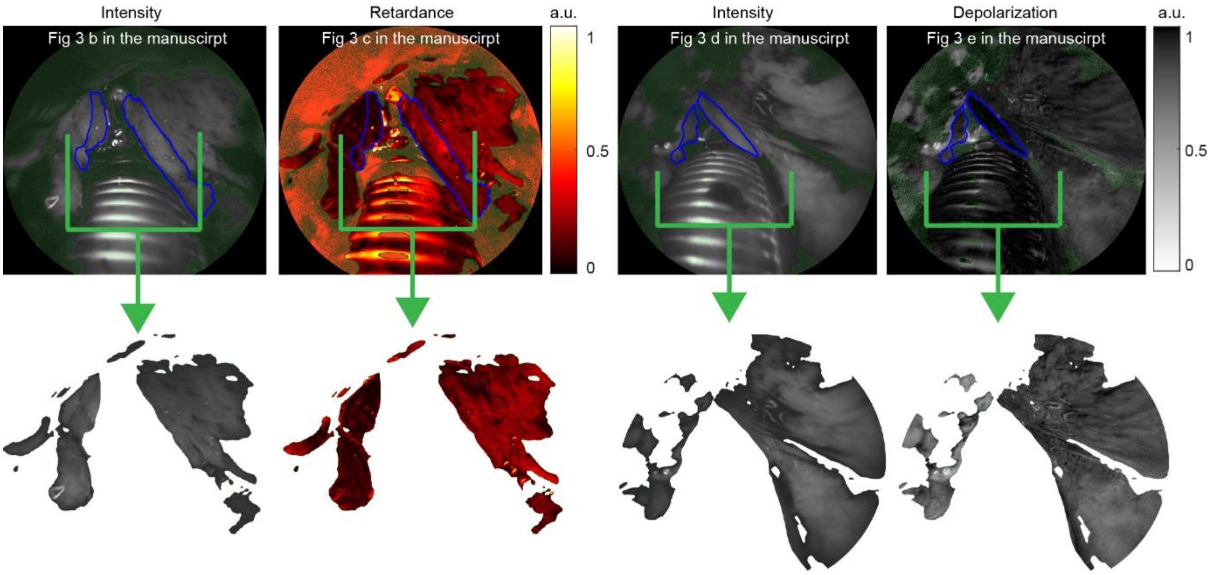

Figure S6. First row: the SPE images of larynx shown in Figure 3(b-e) of the main text. As explained in the main text, some blood that is strongly absorbing re-appeared on the tissue surface during polarimetric endoscopy, resulting in under-exposed regions in the SPE images. Strong specular reflections from smooth tissue surfaces also caused over-exposed/pixel-saturated regions. These regions were rendered green and were treated as invalid and excluded from analysis. Second row: the valid supraglottic regions used for analysis. The under-exposed or over-exposed regions were not shown.

7. Retardance and depolarization images of the *ex vivo* tissue reconstructed from partial Stokes polarimetry

Considering that Mueller matrices contain complete polarization information about tissue, we have restored the partial Stokes polarimetric data from the Mueller polarimetric image of the tissue. Given that the input polarization state is circularly polarized, and the Mueller matrices of the tissue are known, the Stokes vectors of the light emerging from the tissue can be obtained and used to

reconstruct the retardance and depolarization images based on Equation 1-4 specified in the manuscript.

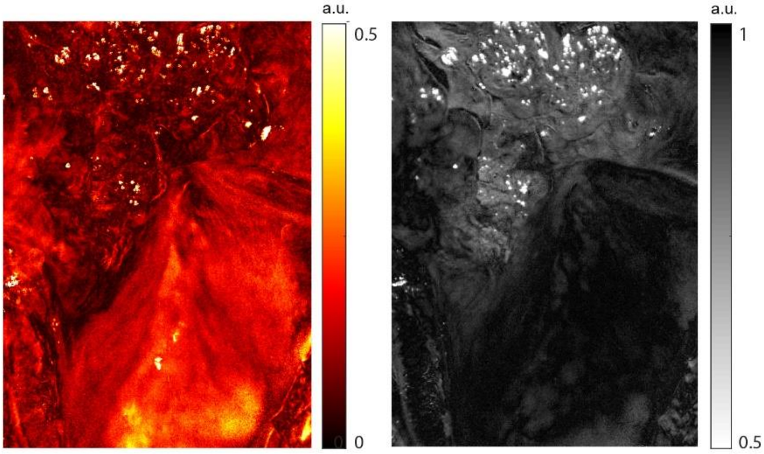

Figure S7. Retardance and depolarization images reconstructed from partial Stokes polarimetry.

8. Receiver operating characteristic (ROC) curves involved in the *ex vivo* tissue experiment

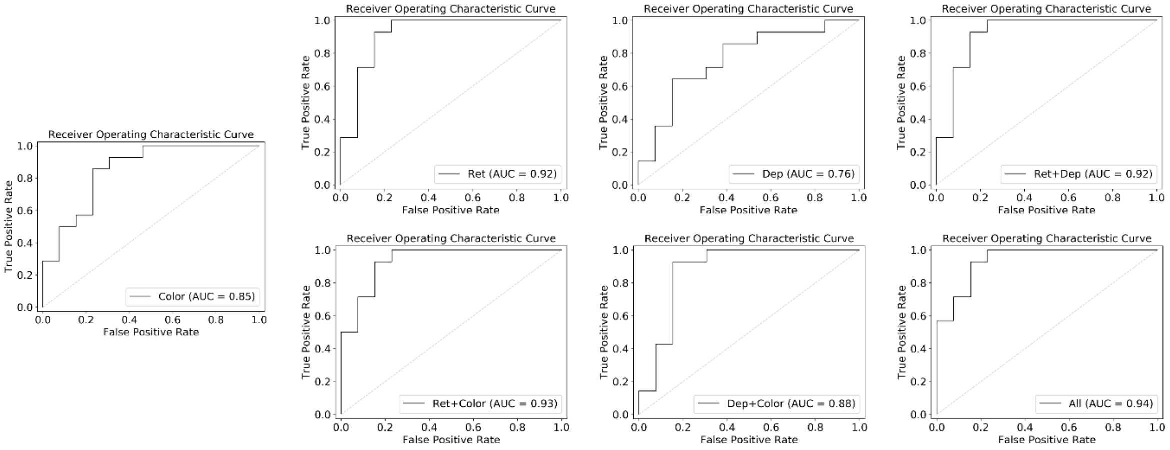

Figure S8. Classification based on Mueller polarimetry.

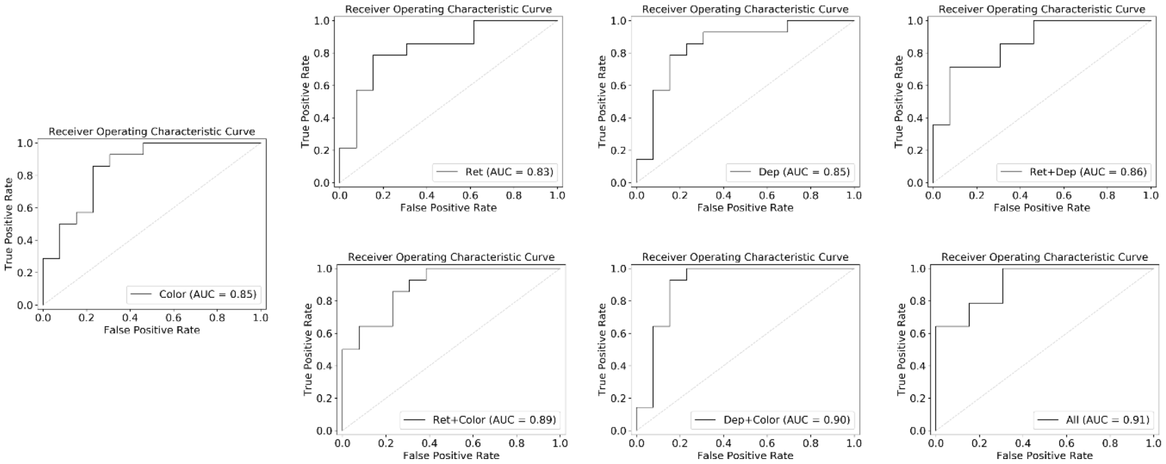

Figure S9. Classification based on partial Stokes polarimetry.

The sensitivity and specificity values for WLE and SPE reported in the paper were the true positive rate and the complement of false positive rate when the maximum of ½ (True Positive Rate + 1 – False

Positive Rate) was reached. It is noted that the above-mentioned sensitivity and specificity were obtained based only on the evaluation of objective information (namely, depolarization, retardance, hue and saturation values) given by polarimetric endoscopy and white light endoscopy. The subjective information which is difficult to quantify was not utilized, e.g. tissue surface textures and anatomical symmetry visible under SPE and WLE. The actual sensitivity and specificity for the proposed method are expected to increase when all the information is exploited.

9. Evaluating the influence of the perspective viewing angle and tissue surface shape

We have conducted additional complementary experiments to evaluate the influence of the perspective viewing angle and tissue surface shape respectively.

I. Viewing perspective angle

We first evaluated the influence of the viewing perspective angle to the SPE retardance images. A turbid phantom with retardance contrast (Phantom 1, see Figure S10a) was fabricated and imaged from different viewing perspective angles under the retardance mode. Phantom 1 has three layers: layer 1 (top) consisted of intralipid solution to mimic the non-retarding laryngeal epithelium; layer 2 (middle) **either** embedded retarding film mimicking the birefringent lamina propria, **or** non-retarding layer, mimicking cancerous tissues; layer 3 (bottom) scattering and depolarising layer mimicking scattering and depolarization in deeper tissue. The viewing perspective angle was changed from 0 deg (viewing direction parallel to the surface normal) to 40 deg in steps of 5 deg. The results are shown in Figure S10.

It was found that when the viewing perspective increased: (1) the non-retarding part of the phantom retained low retardance values; (2) the retarding part still demonstrated higher retardance values than the non-retarding parts; (3) although the retardance values of the retarding part slightly reduced at larger viewing perspectives, they were still higher than that of the non-retarding part obtained for any perspective; (4) the contrast between the non-retarding part and the retarding part does not disappear and could be differentiated based on the information obtained from SPE-retardance images. Although the viewing perspective did influence the retardance values, it would not affect binary classification of the retarding targets from non-retarding targets.

For completeness, Phantom 1 was also imaged under the depolarization mode of SPE. The retarding part of the phantom had slightly higher depolarization than the non-retarding background, because we employed a simplified approach to reconstruct retardance and depolarization from partial Stokes polarimetry. This approach is pragmatic but not as rigorous as Mueller polarimetry in terms of decoupling depolarization and retardance, as discussed in the main text. When the viewing perspective increased, the depolarization values for both the retarding and non-retarding parts rose slightly. Considering that the normal larynx tissues are more likely to associate with high retardance and high depolarization (while in general the tumorous tissues correspond to low retardance and low depolarization), imaging of the normal tissue with increased viewing perspective may lead to reduced retardance contrast, but slightly increased depolarization difference from tumorous tissue, and vice-versa. Since the classification is based on both retardance and depolarization information, the classification results will not be significantly affected by the viewing perspective.

A similar phantom experiment evaluated the influence of the viewing perspective on SPE-depolarization. A turbid phantom was fabricated with depolarization contrast (Phantom 2, see Figure S10a below). This phantom had intralipid solution in two adjacent wells in a well-plate (see Figure S10 a). The solution in the top well included an absorber (ink), demonstrating smaller depolarization than the bottom well. The perspective of the SPE was changed from 0 deg to 40 deg with a step of 5 deg.

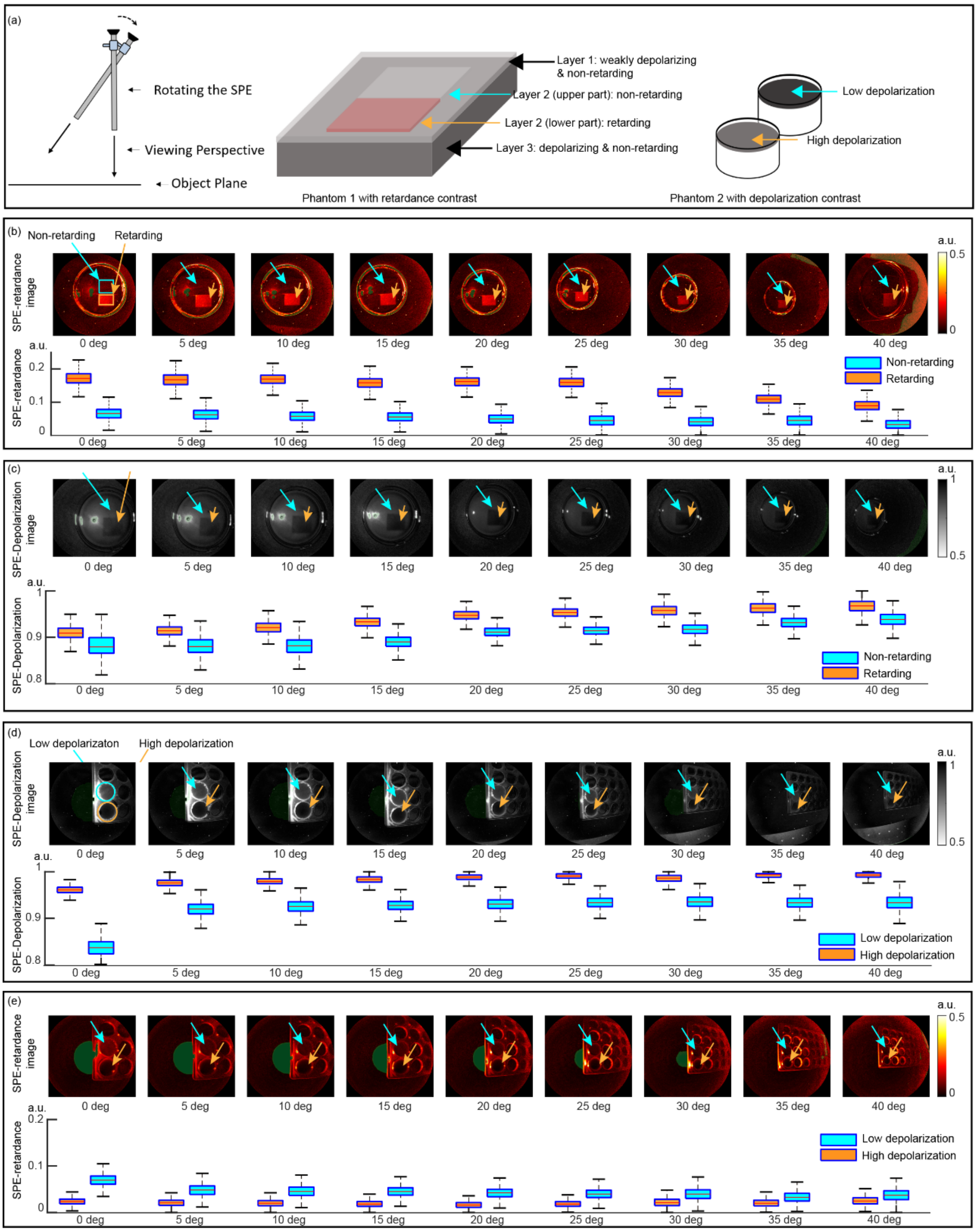

Figure S10. (a) The experimental set-up and phantom structure to determine the effect of changes of perspective; (b) evaluation of SPE-retardance for Phantom 1 from different viewing perspective angles (n= 14553, 9706, 13764, 13108, 9154, 9487, 8164, 6624, 5461 pixels for the non-retarding part and 19339, 9540, 18648, 14490, 14112, 10556, 8216, 6165, 6000 pixels for the retarding part respectively). (c) evaluation of SPE-depolarization for Phantom 1 from different viewing perspective angles (n= 10620, 10912, 7038, 8030, 6351, 5236, 4838, 2340, 12276 pixels for the non-retarding part and 10005, 9605, 7245, 7004, 5040, 4760, 3726, 2360, 5520 for the retarding part respectively). (d) evaluation of SPE-depolarization for Phantom 2 from different viewing perspective angles (n = 10890, 10192, 9207, 6804, 6351, 4899, 4284, 2652, 1836 pixels for the part of low depolarization and 7031, 8400, 7189, 7905, 6853, 4347, 2912, 2754, 1326 pixels for the part of high depolarization respectively). (e) evaluation of SPE- depolarization for Phantom 2 from different viewing perspective angles (n = 5740, 6536, 6090, 5610, 5700, 4080, 3050, 2236, 1804 pixels for the of low depolarization and 7826, 6825, 7280, 5040, 4347, 3705, 2940, 2652, 1936 for the part of high depolarization respectively). In the boxplots presented in this figure, the red center line denotes median and the blue box shows the 25th and 75th percentiles of the dataset. The black whiskers mark the non-outlier minimum and non-outlier maximum.

As the viewing perspective increased, the depolarization contrast between the wells was retained (see Figure S10d) and the two wells could be differentiated for all these perspectives. This suggests that the depolarization contrast reported in the manuscript would not disappear if the viewing perspective of the SPE changed and can still be useful to classify the tissue.

In fact, there is not much variation of viewing perspective during the surgical procedure since the angle of the endoscope is confined by a narrow (typically 2 cm diameter) and long (around 15 cm) cannula (see Figure S11), which is rigid and securely fixed to patient's body. The results from this additional experiment suggest that the polarimetric contrast between the cancerous tissue and the normal tissue reported in the manuscript would not disappear even if the perspective of the SPE slightly changed and is still useful for tissue classification.

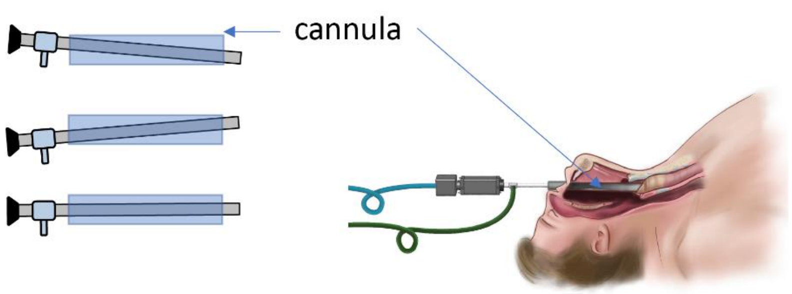

Figure S11. The viewing perspective angle of an endoscope is confined by a narrow and long cannula in surgery.

II. Tissue surface shape

An arbitrary surface can be approximated as consisting of many small flat microfacets with different surface normals, which effectively makes this scenario equivalent to viewing a horizontal flat surface from different viewing perspective angles. Therefore, the influence of the shape to polarization signals is essentially the same as that of the viewing perspective angle evaluated in the previous section. Therefore, as above, although surface shape may affect the quantitative polarimetric values, the polarimetric contrast between the cancerous tissue and the normal tissue should remain.

We conducted an additional experiment to assess the influence of shape by imaging a curved highly scattering, depolarizing, homogeneous, low retardance white paper target (its shape is approximately a circular segment with 2.5 cm sagitta and 10 cm chord length, see Figure S12a). A subjective assessment of the intensity, retardance and depolarization images shown in Figure S12b indicated that the intensity is most strongly affected by the shape, while the retardance and depolarization are minimally affected. This is quantified in the line profiles in Figure S12c, where the maximum minus the minimum values of the three line profiles are 0.389, 0.045, 0.020 for intensity reference (normalized to 0-1), depolarization and retardance respectively, indicating the influence of shape on polarization is relatively small. We also note that the regions where depolarization is slightly increased had a surface normal parallel to the endoscope where reflections are dominated by specular highlights for larynx tissues and are likely to be removed from processing (for example, in this work, we used a green mask to cover these invalid areas).

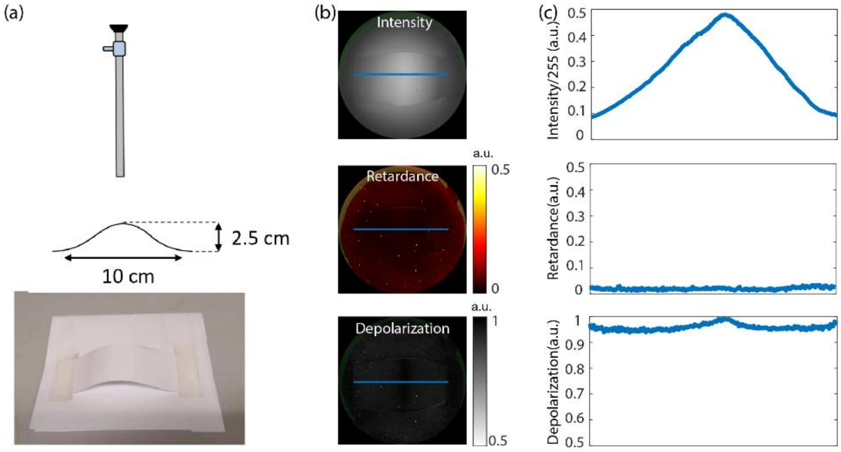

Figure S12. (a) The imaging target of white paper formed into an arc; (b) intensity, retardance and depolarization imaging of the target; (c) the intensity, retardance and depolarization profiles corresponding to the blue lines in (b).

We additionally reviewed the polarized light scattering spectroscopy (PLSS) literature (3-7). PLSS uses co-polarized and cross-polarised light to obtain approximate depolarization (ignoring any tissue anisotropy) and is used for cell morphological analysis. PLSS should entail higher precision where the influence of shape and the viewing direction might be necessary because cell morphology had to be quantified from the model-based inversion process. In comparison, SPE uses polarimetric contrast for binary categorical classification, which is qualitative rather than quantitative, and the requirement of the precision would be relatively low. To the best of our knowledge, tissue surface shape and the viewing perspective angle of the probe were not considered in the PLSS model and its inversion process (3, 4), but the technique still achieved excellent Barrett's-associated dysplasia diagnosis (92% sensitivity and 96% specificity) and malignant pancreatic cyst assessment (90% sensitivity and 100% specificity) (3, 4).

In summary, based on the additional experiments, considering how SPE would be used in practice, and after reviewing the literature for similar tissue diagnosis, we believe these factors will not prevent SPE from being used for tissue classification.

10. Virtual white light endoscopy (WLE) with SPE via deep learning

Given that using color and polarimetric information together can yield better performance, it would be of value to integrate these two modalities into one device. The current SPE system used narrow band illumination and DoFP-LP image sensor which does not have a color Bayer filter array but a micro-linear polarizer array on top of the photo-detector array. Therefore, it does not provide an image equivalent to the color image under white light, but a monochrome grayscale intensity reference image besides retardance or depolarization images. Here, we demonstrate that a virtual WLE technique for SPE, which transforms the monochrome intensity reference image obtained with the SPE system to a WLE image via deep learning.

We used a generative adversarial network (GAN) framework (8, 9), which turns out suitable for image generation and style transfer tasks without blurry artefacts, to learn the transformation from an intensity reference image to the corresponding WLE image. Training a GAN involves two different networks, namely, a generator and a discriminator, shown in Figure S13. The network architecture of the generator follows that of U-net to perform the transformation (10), which is capable of preserving high spatial frequency information within the input image in a encoder-decoder network via adding skip connections. A discriminator follows a standard convolutional neural network architecture to

learn a criterion that aims to accurately classify whether the generated virtual WLE image is real or fake and reject those output inconsistent with the label during training. The loss functions for the generator and discriminator were defined as such,

$$L_g = E\{-\log(\mathrm{sigmoid}(D(\mathrm{I}_{\mathrm{input}}, \mathrm{I}_{\mathrm{generated}})))\} + \lambda \times \left\| \mathrm{I}_{\mathrm{label}} - \mathrm{I}_{\mathrm{generated}} \right\|_{l1}$$

$$L_d = E\{-\log(\mathrm{sigmoid}(D(\mathrm{I}_{\mathrm{input}}, \mathrm{I}_{\mathrm{generated}})))-\log(1-\mathrm{sigmoid}(D(\mathrm{I}_{\mathrm{input}}, \mathrm{I}_{\mathrm{label}})))\}$$

where G, D, I_{input}, $I_{\mathrm{generated}}$, I_{label}, λ, E denote generator, discriminator, the input intensity reference image from SPE, the generated virtual WLE image, the ground truth WLE image in the training dataset, , the regularization parameter and the expectation respectively. The convolutional kernels throughout the GAN network were set to be 5×5, and were randomly initialized. λ was determined as 100 empirically. The learnable parameters in the neural network were updated through the training stage using an adaptive moment estimation (Adam) optimizer with a learning rate of 0.002. For each training step (or iteration) of the discriminator network, there were two training steps of the generator network to avoid training stagnation arising from an overfit of the discriminator and an underfit of the generator.

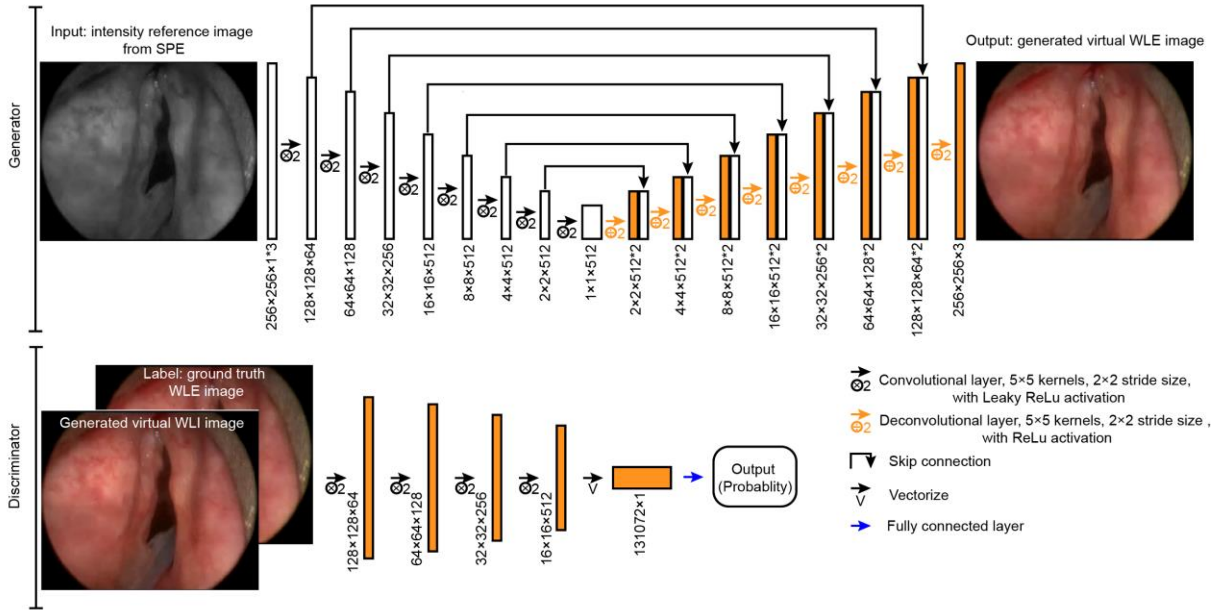

Figure S13 Deep neural network architecture (generative adversarial network (GAN)) for virtual WLE during SPE. The generator of the GAN is a U-net including 7 convolutional and 7 deconvolutional layers with skip connections, and converts intensity reference images from SPE to the virtual WLE images. The discriminator is a standard convolutional neural network with 4 convolutional layers and a fully connected layer, learns a criterion that aims to accurately classify whether the generated virtual WLE image is real or fake in the form of probability and reject those output inconsistent with the label during training. The layers are represented by white and orange blocks in the figure. The convolutional kernel size and stride size throughout the GAN network were 5×5 and 2×2 respectively.

Four real WLE video clips (each last about 1 minute) including two from the laryngectomy case, and the other two from the transoral surgery case were used for training. The video frames were then converted into individual color images (as label images for training and ground truth for testing). To increase the diversity of data available for training so as to reduce overfitting, data were augmented via random flipping, rotation (-10 deg to 10 deg), scaling and cropping. The augmented color images were then converted to grayscale images as the networking input for training and testing, and were

randomly split into two datasets with one for training (2000 images) and the other for testing (500 images). Data preparation and augmentation was implemented with OpenCV 4.0.2 and Python-imgaug 0.4.0. Training and testing of the network were implemented using Python 3.6 with Tensorflow 1.6.0.

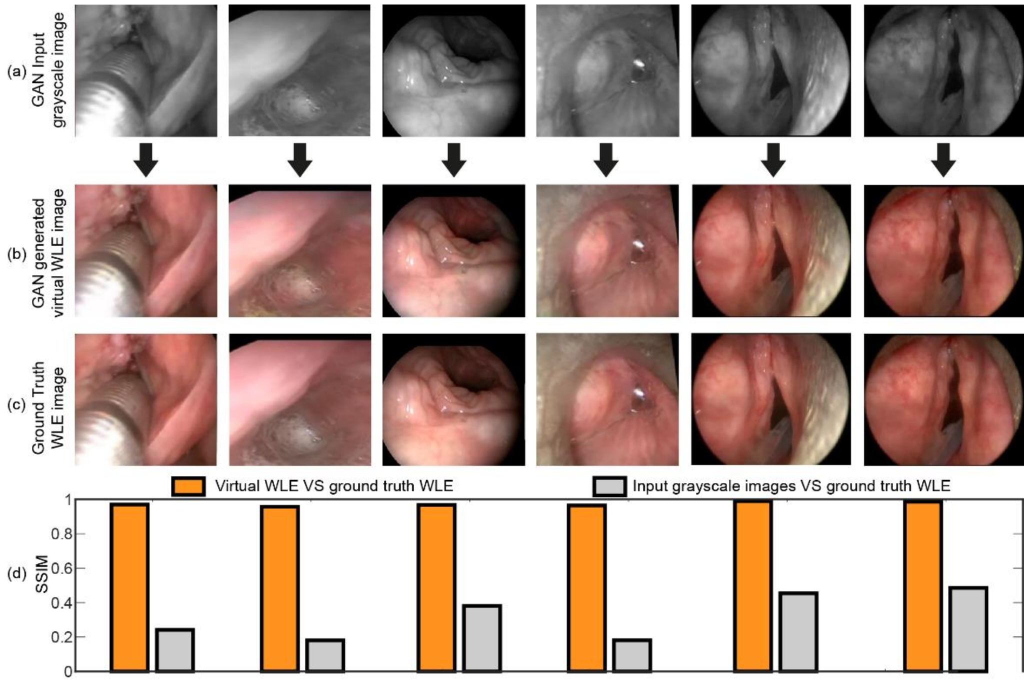

Figure S14 Testing of the network for virtual WLE. (a) the input of the network are grayscale images corresponding to the monochrome intensity reference images in SPE, (b) the output generated virtual WLE images, (c) ground truth WLE images, (d) Structural Similarity indices (SSIM) between the virtual WLE and the ground truth WLE, and those between the ground truth WLE and the input grayscale images (the red-green-blue channels have equal values) for comparison.

After training for 20 epochs with the training dataset, the virtual WLE network was tested with the test dataset. As shown in Figure S14, the virtual WLE and the ground truth WLE images were difficult to distinguish in general via subjective assessment. We used structural similarity index (SSIM) ranging from 0 (no similarity) and 1 (completely equivalent) to objectively characterize the similarity between the virtual WLE and the ground truth WLE. The mean of the SSIM reached 0.973, close to being equivalent, indicating the generated virtual WLE is highly similar to the ground truth. For comparison, we calculated the SSIM between the ground truth WLE and the input grayscale image (the red-green-blue channels have equal values). The mean of the SSIM is 0.330. This indicated the network can effectively increase the similarity for virtual WLE in SPE.

The virtual WLE images were then converted from the intensity reference images from intraoperative SPE, demonstrated in Figure 3 and Figure 5 in the main text, shown in Figure S15. Therefore, it is feasible to extend the current SPE system to simultaneously provide color information about the tissue, in addition to the intensity reference and polarimetric images.

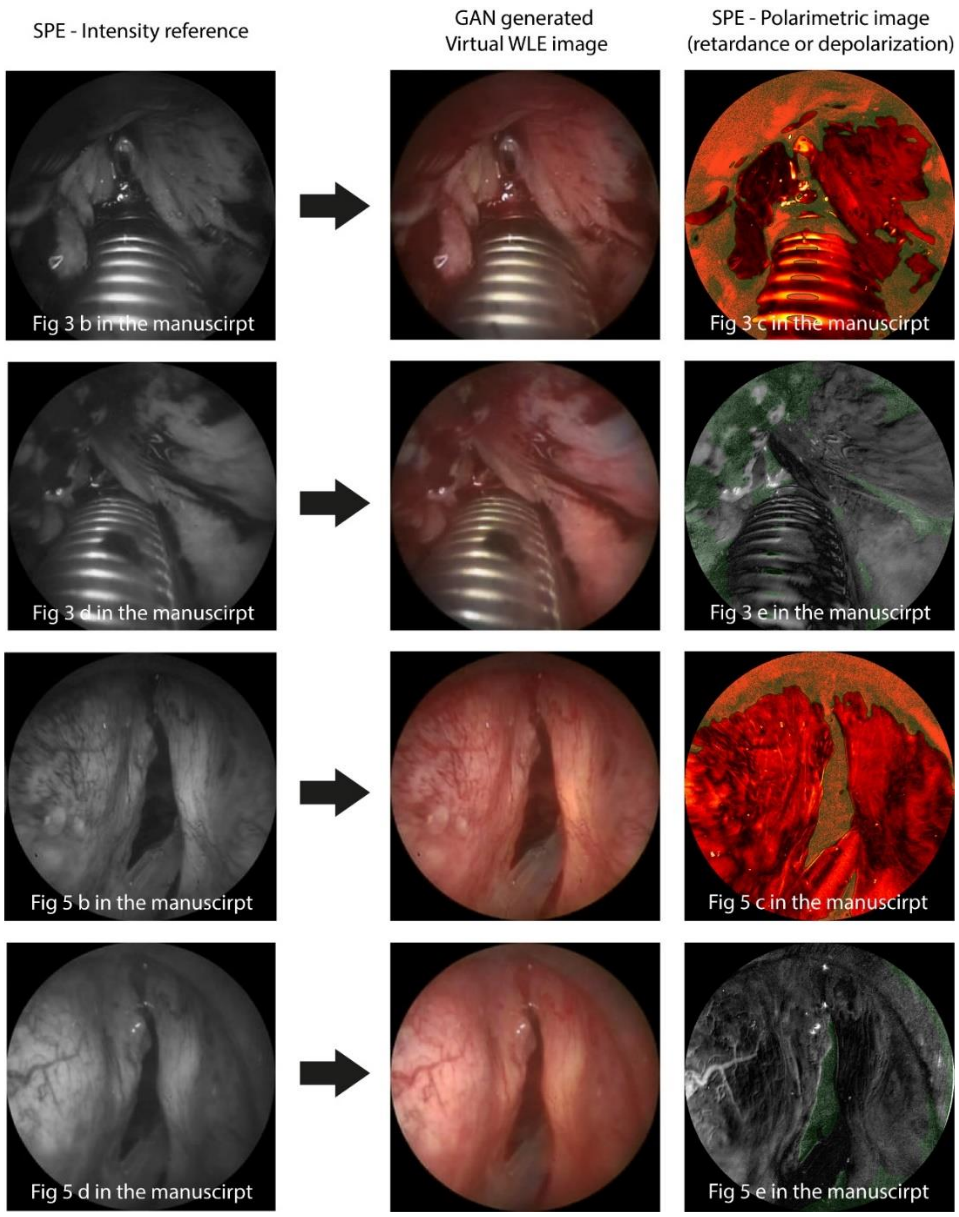

Figure S15 Virtual WLE images converted from the intensity references images which were acquired during intraoperative SPE *in vivo* by the GAN deep neural network. The first column of images are the intensity references shown in Fig 3b, Fig 3d, Fig 5b and Fig 5d in the main text respectively; the second column are virtual WLE images generated by GAN; the third column are the corresponding polarimetric images shown in Fig 3c, Fig 3e, Fig 5c and Fig 5e in the main text respectively.

11. Polarization image regions for retardance index calculation.

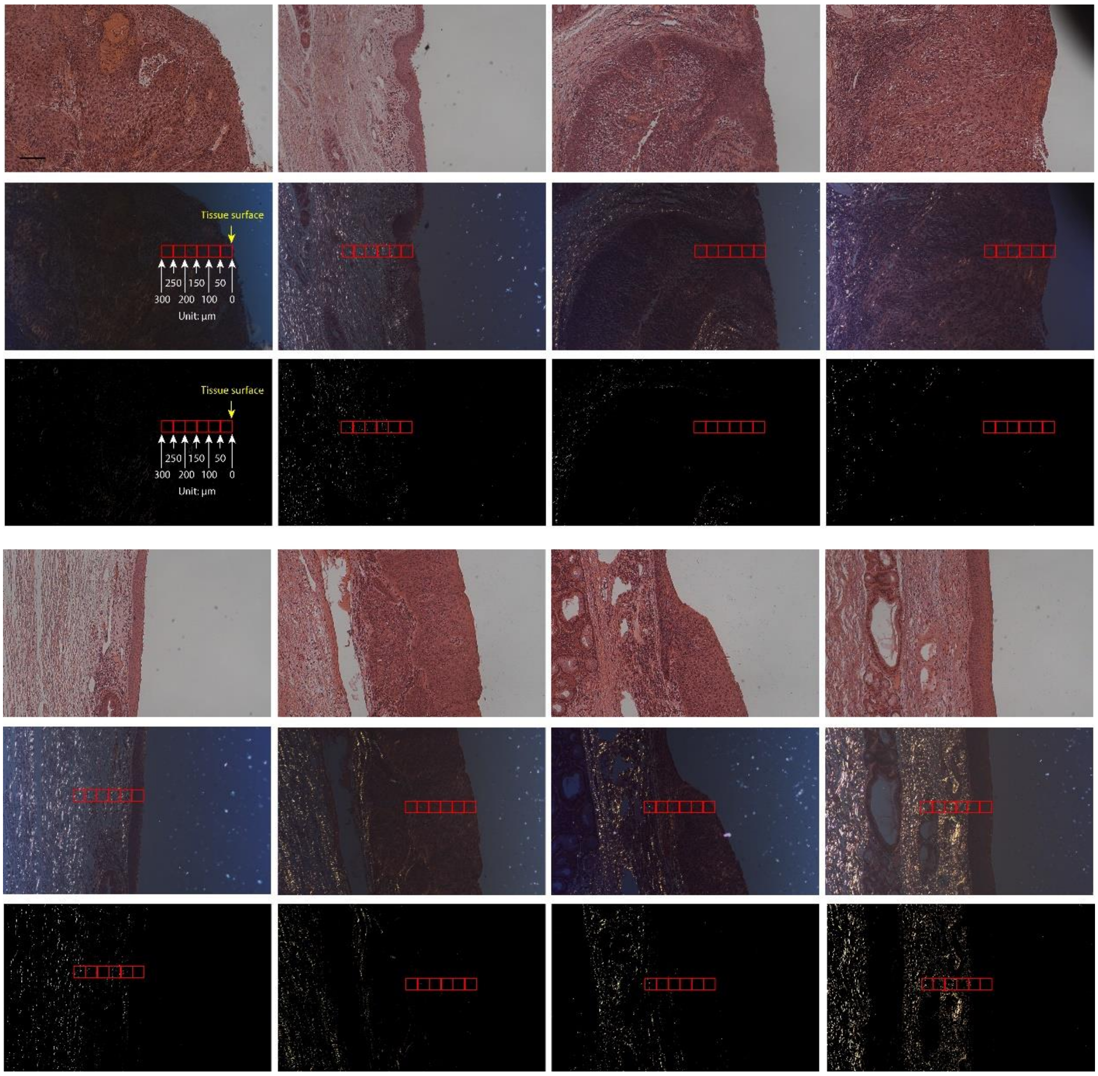

Figure S16 Regions for retardance index calculation during polarization microscopy image analysis. First row: the routine H&E images of ROI 1-4 in Figure 4 in the main text; Second row: the polarization microscopy images of ROI 1-4; Third row: birefringent pixels extracted from the polarization microscopy images. The non-birefringent pixels are dark. The overlaid red boxes refer to those regions to calculate the retardance index. The individual red boxes from right to left represents the most superficial 0-50 um, 50-100 um, 100-150 um, 150-200 um, 200-250 um and 250-300 um tissue volume. These regions were located by manually reading the coordinates of tissue surfaces in each slice.

12. Using SPE to differentiate cancerous from inflammatory lesions: feasibility analysis

It is clinically desirable to differentiate cancerous verses inflammatory lesions. Based on the literature and the findings reported in the manuscript, we briefly analyse the feasibility of using SPE to achieve this goal. In cancerous lesions, uncontrolled cancer cell proliferation disrupts normal tissue architectures by destroying the structure of the non-retarding epithelium and invading the retarding lamina propria, resulting in a low retardance value, as reported in the manuscript. Inflammation (induced by smoking for example) is usually associated with oedema, hyperaemia and increased number of granulocytes and mononuclear cells, but the retarding lamina propria in the tissue is still in place and the general tissue architectures typically maintain a normal order (11, 12). As a result, the retardance of the inflammatory tissues should be higher than the cancerous lesions (but lower than normal tissue due to reduced density and order of the retarding structures in the lamina propria resulting from oedema). Due to increased absorption arising from hyperaemia, it is expected that

depolarization in the inflammatory tissues should be weak. Cancerous lesions are also expected to have weak depolarization due to enhanced vascularization and destroyed tissue architectures. In the following table, we summarise the anticipated polarimetric properties of cancer/dysplasia, normal and inflammatory tissues. SPE should therefore have potential to differentiate cancerous versus inflammatory lesions.

	**Cancerous**	**Normal**	**Inflammatory**
**Retardance**	Low	High	Moderate/High
Histological Origin	*Uncontrolled cancer cell proliferation destroys the structure of non-retarding epithelium and invading retarding lamina propria.*	*Retarding lamina propria is intact.*	*Lamina propria with reduced retarding structures is still intact.*
**Depolarization**	Low	High	Low
Histological Origin	*Enhanced vascularization of the tumour and reduction in scattering due to the destruction of normal tissue architectures.*	*Blood supply and tissue architectures are in good order.*	*Hyperaemia.*

13. Supplementary video information

Areas rendered green in the videos below are either under- or over-exposed.

Supplementary Video 1

Consecutive SPE-retardance imaging of the volunteer's oral vestibule *in vivo*

Left: intensity reference images

Right: retardance images

Supplementary Video 2

Consecutive SPE-depolarization imaging of the volunteer's oral vestibule *in vivo*

Left: intensity reference images

Right: depolarization images

Supplementary Video 3

Consecutive SPE-retardance imaging of the patient requiring laryngectomy *in vivo*

Left: intensity reference images

Right: retardance images

Supplementary Video 4

Consecutive SPE-depolarization imaging of the patient requiring laryngectomy *in vivo*

Left: intensity reference images

Right: depolarization images

Supplementary Video 5

Consecutive SPE-retardance imaging of the patient requiring transoral surgery *in vivo*

Left: intensity reference images

Right: retardance images

Supplementary Video 6

Consecutive SPE-depolarization imaging of the patient requiring transoral surgery *in vivo*

Left: intensity reference images

Right: depolarization images

We labelled the anatomical position for all the frames of Video 3-6 and the key landmarks (1-glottis and 2-supraglottis for video 3 & 4; 1-anterior vocal cord and 2-posterior vocal cord for video 5 & 6) in their first ten frames. The white light images, the polarimetric images and their intensity reference corresponding to the scene involved in Video 3-6 (also demonstrated in Figure 3(a-e) and Figure 5(a-e) in the manuscript) were also provided with labels as a reference.

14. References

1. Compain E, Poirier S, Drevillon B. General and Self-Consistent Method for the Calibration of Polarization Modulators, Polarimeters, and Mueller-Matrix Ellipsometers. Appl Opt. 1999;38(16):3490-502.
2. Lu S-Y, Chipman RA. Interpretation of Mueller matrices based on polar decomposition. J Opt Soc Am A. 1996;13(5):1106-13.
3. Qiu L, Pleskow DK, Chuttani R, Vitkin E, Leyden J, Ozden N, et al. Multispectral scanning during endoscopy guides biopsy of dysplasia in Barrett's esophagus. Nat Med. 2010;16(5):603-6.
4. Zhang L, Pleskow DK, Turzhitsky V, Yee EU, Berzin TM, Sawhney M, et al. Light scattering spectroscopy identifies the malignant potential of pancreatic cysts during endoscopy. Nature Biomedical Engineering. 2017;1(4):0040.
5. Qiu L, Chuttani R, Pleskow DK, Turzhitsky V, Khan U, Zakharov YN, et al. Multispectral light scattering endoscopic imaging of esophageal precancer. Light: Science & Applications. 2018;7:17174.
6. Backman V, Gurjar R, Badizadegan K, Itzkan I, Dasari RR, Perelman LT, et al. Polarized light scattering spectroscopy for quantitative measurement of epithelial cellular structures in situ. IEEE J Sel Top Quantum Electron. 1999;5(4):1019-26.
7. Gurjar RS, Backman V, Perelman LT, Georgakoudi I, Badizadegan K, Itzkan I, et al. Imaging human epithelial properties with polarized light-scattering spectroscopy. Nat Med. 2001;7(11):1245-8.
8. Goodfellow I, Pouget-Abadie J, Mirza M, Xu B, Warde-Farley D, Ozair S, et al., editors. Generative adversarial nets. Advances in neural information processing systems; 2014.

9. Isola P, Zhu J-Y, Zhou T, Efros AA, editors. Image-to-image translation with conditional adversarial networks. Proceedings of the IEEE conference on computer vision and pattern recognition; 2017.
10. Ronneberger O, Fischer P, Brox T, editors. U-net: Convolutional networks for biomedical image segmentation. International Conference on Medical image computing and computer-assisted intervention; 2015: Springer.
11. Liu X, Mustonen A, Zheng W, Sivasankar MP, Durkes AC. Cigarette smoke exposure to pig larynx in an inhalation chamber. Journal of Voice. 2019;33(6):846-50.
12. Mouadeb DA, Belafsky PC, Birchall M, Hood C, Konia T, Pinkerton KE. The effects of allergens and tobacco smoke on the laryngeal mucosa of guinea pigs. Otolaryngology—Head and Neck Surgery. 2009;140(4):493-7.
