# Supplementary material for: Surgical polarimetric endoscopy for the detection of laryngeal cancer
Source: Nat Biomed Eng. 2023 Apr 3;7(8):971–85. doi: 10.1038/s41551-023-01018-0 (PMC10427430; doi:10.1038/s41551-023-01018-0)
Supplement: Supplementary file 2 — Reporting Summary [file 41551_2023_1018_MOESM2_ESM.pdf]

nature research

Corresponding author(s): Ji Qi, Danail Stoyanov, Daniel Elson

Last updated by author(s): Dec 10, 2022

Reporting Summary

Nature Research wishes to improve the reproducibility of the work that we publish. This form provides structure for consistency and transparency in reporting. For further information on Nature Research policies, see our Editorial Policies and the Editorial Policy Checklist.

Statistics

For all statistical analyses, confirm that the following items are present in the figure legend, table legend, main text, or Methods section.

n/a	Confirmed	
☐	☒	The exact sample size (n) for each experimental group/condition, given as a discrete number and unit of measurement
☐	☒	A statement on whether measurements were taken from distinct samples or whether the same sample was measured repeatedly
☐	☒	The statistical test(s) used AND whether they are one- or two-sided<br>*Only common tests should be described solely by name; describe more complex techniques in the Methods section.*
☒	☐	A description of all covariates tested
☒	☐	A description of any assumptions or corrections, such as tests of normality and adjustment for multiple comparisons
☐	☒	A full description of the statistical parameters including central tendency (e.g. means) or other basic estimates (e.g. regression coefficient) AND variation (e.g. standard deviation) or associated estimates of uncertainty (e.g. confidence intervals)
☐	☒	For null hypothesis testing, the test statistic (e.g. F, t, r) with confidence intervals, effect sizes, degrees of freedom and P value noted<br>*Give P values as exact values whenever suitable.*
☒	☐	For Bayesian analysis, information on the choice of priors and Markov chain Monte Carlo settings
☒	☐	For hierarchical and complex designs, identification of the appropriate level for tests and full reporting of outcomes
☒	☐	Estimates of effect sizes (e.g. Cohen's d, Pearson's r), indicating how they were calculated

Our web collection on statistics for biologists contains articles on many of the points above.

Software and code

Policy information about availability of computer code

Data collection	SPE data were collected using a LabVIEW2017 program.
Data analysis	We used MATLAB 2017 for image analysis, statistical analysis, data visualization; Python 3.7.4, with scikit-learn 0.21.3 for classification; Python 3.6.8 with Tensorflow 1.6.0, OpenCV 4.0.2 and Python-imgaug 0.4.0.for the work related to virtual white-light endoscopy. The custom code is available on the Zenodo repository at https://doi.org/10.5281/zenodo.7435189.

For manuscripts utilizing custom algorithms or software that are central to the research but not yet described in published literature, software must be made available to editors and reviewers. We strongly encourage code deposition in a community repository (e.g. GitHub). See the Nature Research guidelines for submitting code & software for further information.

Data

Policy information about availability of data

All manuscripts must include a data availability statement. This statement should provide the following information, where applicable:

- Accession codes, unique identifiers, or web links for publicly available datasets
- A list of figures that have associated raw data
- A description of any restrictions on data availability

The data supporting the results in this study are available within the paper and its Supplementary Information. Source data for the figures are provided with this paper. The raw images involving human participants are protected owing to data-privacy requirements, and can be made available for research purposes on reasonable request from the corresponding authors, provided that approval is obtained after an institutional review procedure at Imperial College London and London North West University Healthcare NHS Trust. The response from the authors will usually be within four weeks.

Field-specific reporting

Please select the one below that is the best fit for your research. If you are not sure, read the appropriate sections before making your selection.

☒ Life sciences ☐ Behavioural & social sciences ☐ Ecological, evolutionary & environmental sciences

For a reference copy of the document with all sections, see nature.com/documents/nr-reporting-summary-flat.pdf

Life sciences study design

All studies must disclose on these points even when the disclosure is negative.

Sample size	We did not conduct a formal power analysis to determine the sample size, owing to the absence of a priori mean and standard deviation polarimetric values for the larynx and thereby the effect size. We aimed to collect as many samples as was reasonably possible given the experimental constraints. A post-hoc analysis has been informally conducted based on the mean and standard deviation obtained in this study using the G*Power 3.1 software [Faul, F., et. al. Behav. Res. Methods, 41.], and given two-sided Mann-Whitney test, α=0.01 and power=0.99, the sample size was estimated to be 34 and 21 for the evaluation of retardance and depolarization respectively. The sample sizes in this study are therefore sufficient according to this analysis.
Data exclusions	No data were excluded. Underexposed regions (that is, caused by blood on the tissue surface) and overexposed regions (that is, with strong specular reflection) in the SPE images were marked (rendered green with transparency) in display to avoid misleading viewers. These regions were invalid and not used in data analysis, as clearly stated in the paper. The criteria for underexposure and overexposure were pre-established upon the greyscale of polarization-insensitive-intensity reference image, and are clearly stated in Methods.
Replication	The patient requiring laryngectomy was consecutively imaged 28 times under the retardance mode and 34 times under the depolarization mode. Each time the larynx was imaged from a different view and working distance, owing to the motion of the tissue and the endoscope. The polarimetric properties of the normal and tumorous tissue and their polarimetric contrast remained consistent, and are included in the Supplementary videos. The findings from the first patient were reproduced in the second patient, who was consecutively imaged 30 times under the retardance mode and 71 times under the depolarization mode, respectively. The consistent polarimetric properties are included in the Supplementary videos. The results obtained in vivo also agreed with those obtained ex vivo as well as during histology and polarization-microscopy validation.
Randomization	Randomization was not required, because all the samples underwent both polarimetric imaging (the method being evaluated) and white-light imaging (the control method), and were compared with pathology (the gold-standard), thereby eliminating potential bias when comparing the two methods in this observational study. This is consistent with other medical-imaging technology evaluation and validation studies, according to [P. Valk, J. Nucl. Med. 41,7; and, E. Lalumera & S. Fanti, Topoi 38, 2.].
Blinding	The pathologist who processed the resected tissue samples was blind to images obtained from SPE.

Reporting for specific materials, systems and methods

We require information from authors about some types of materials, experimental systems and methods used in many studies. Here, indicate whether each material, system or method listed is relevant to your study. If you are not sure if a list item applies to your research, read the appropriate section before selecting a response.

Materials & experimental systems

n/a	Involved in the study	
☒	☐	Antibodies
☒	☐	Eukaryotic cell lines
☒	☐	Palaeontology and archaeology
☒	☐	Animals and other organisms
☐	☒	Human research participants
☒	☐	Clinical data
☒	☐	Dual use research of concern

Methods

n/a	Involved in the study	
☒	☐	ChIP-seq
☒	☐	Flow cytometry
☒	☐	MRI-based neuroimaging

Human research participants

Policy information about studies involving human research participants

Population characteristics	The patient requiring laryngectomy was a 64-year-old female, presenting with dysphonia and stridor, radiologically staged T4N0 squamous cell carcinoma and confirmed via routine endoscopy and biopsy 6 weeks before the laryngectomy surgery. Total laryngectomy was planned as the main treatment. The patient undergoing transoral surgery was a 63-year-old male, presenting with dysphonia. Routine fiber-optic nasendoscopy revealed left glottic lesion. Biopsy was planned for diagnosis. Transoral surgery was performed in the same procedure with biopsy.
Recruitment	Patient requirement for the intraoperative study was conducted in Northwick Park Hospital, London North West University

Recruitment	Healthcare NHS Trust, UK between December 2016 and January 2017. Inclusion criteria of recruitment included: (1) laryngeal cancer diagnosed, and planned to receive tumour-resection surgery, older than 18 years and younger than 80, informed consent signed; or (2) suspect laryngeal cancer lesions noted in earlier examination, and planned to receive biopsy, followed with or without surgical resection, older than 18 years and younger than 80, informed consent signed. The investigators randomly recruited the participants with detailed explanations of the purposes and possible risks for this observational study. The application scenario of polarimetric endoscopy would be used for people with laryngeal cancer diagnosed or with suspect laryngeal cancer lesions as a complement to white-light endoscopy, so these inclusion criteria is unlikely to impact the results. The age criterion covers about 94.4% of the incidence (using the statistics in New York State as an example https://www.health.ny.gov/statistics/cancer/registry/table6/tb6larynxbronx.htm), so it is unlikely to impact the results significantly.<br><br>Volunteer recruitment for the validation study was conducted in Imperial College London in the UK in December 2019. Inclusion criteria of recruitment were a healthy adult, older than 18 years and younger than 80, informed consent signed. The investigators randomly recruited the participants with detailed explanations of the benefits and possible risks. The experiment was mainly designed to assess the technical performance of SPE. The inclusion criteria are unlikely to impact the results.
Ethics oversight	This clinical study (intraoperative in vivo imaging, ex vivo tissue imaging, microscopy) was approved by NHS Central London Research Ethics Committees (reference number 10/H0718/55). The technical validation study (oral cavity and skin imaging in vivo) was approved by the Joint Research Compliance Office Imperial College London under the Science Engineering Technology Research Ethics Committee process (reference number 20IC5863). Informed consent was obtained from the patients.

Note that full information on the approval of the study protocol must also be provided in the manuscript.
